# Transcriptional landscape of highly lignified poplar stems at single-cell resolution

**DOI:** 10.1186/s13059-021-02537-2

**Published:** 2021-11-22

**Authors:** Yang Chen, Shaofei Tong, Yuanzhong Jiang, Fandi Ai, Yanlin Feng, Junlin Zhang, Jue Gong, Jiajia Qin, Yuanyuan Zhang, Yingying Zhu, Jianquan Liu, Tao Ma

**Affiliations:** 1 Key Laboratory of Bio-resource and Eco-Environment of Ministry of Education, College of Life Sciences, Sichuan University, Chengdu, China; 2 State Key Laboratory of Grassland Agro-Ecosystem, Institute of Innovation Ecology, Lanzhou University, Lanzhou, China

## Abstract

**Background:**

Plant secondary growth depends on the activity of the vascular cambium, which produces xylem and phloem. Wood derived from xylem is the most abundant form of biomass globally and has played key socio-economic and subsistence roles throughout human history. However, despite intensive study of vascular development, the full diversity of cell types and the gene networks engaged are still poorly understood.

**Results:**

Here, we have applied an optimized protoplast isolation protocol and RNA sequencing to characterize the high-resolution single-cell transcriptional landscape of highly lignified poplar stems. We identify 20 putative cell clusters with a series of novel cluster-specific marker genes and find that these cells are highly heterogeneous based on the transcriptome. Analysis of these marker genes’ expression dynamics enables reconstruction of the cell differentiation trajectories involved in phloem and xylem development. We find that different cell clusters exhibit distinct patterns of phytohormone responses and emphasize the use of our data to predict potential gene redundancy and identify candidate genes related to vascular development in trees.

**Conclusions:**

These findings establish the transcriptional landscape of major cell types of poplar stems at single-cell resolution and provide a valuable resource for investigating basic principles of vascular cell specification and differentiation in trees.

**Supplementary Information:**

The online version contains supplementary material available at 10.1186/s13059-021-02537-2.

## Background

The evolution of vascular tissue played key roles in plants’ colonization and domination of terrestrial environments by enabling long-distance transport of water and nutrients and providing mechanical strength that supports their vertical growth [1, 2]. These tissues mainly consist of xylem and phloem cells, usually respectively produced by inward and outward division of cells in the vascular cambium, namely a process of secondary growth [3]. A typical feature of this process is thickening of the cell wall in the specialized xylem and phloem cell types, a continuous process of carbon accumulation and generation of the most abundant form of biomass in the world: wood [4]. In recent decades, there has been considerable progress in identifying key enzymes involved in cell wall biosynthesis [5], and a hierarchical regulatory network involving several types of transcription factors that regulate cell specification and differentiation during vascular development has been proposed [6–8]. Those studies were mostly based on roots and inflorescence stems of the model plant *Arabidopsis* [9] and stems of the model tree poplar [10]. However, unlike the herbaceous annual *Arabidopsis*, perennial woody plants exhibit extreme secondary growth, characterized by formation of secondary cell walls (SCW) and additional dynamic seasonal changes influenced by various environmental stresses [11, 12]. Thus, partly due to reliance on information and markers from *Arabidopsis*, the molecular-level regulation of vascular tissue development in trees, especially the SCW regulatory network involved, has not been fully elucidated [10, 13].

Quantifying and comparing gene expression in specific cell types is essential for understanding the complex regulatory networks that control vascular development. By combining use of cell-type-specific reporter lines with cell sorting, laser capture microdissection, microarray analysis, and RNA sequencing, extensive studies have characterized the gene expression patterns of certain types of vascular cells in poplars, such as fusiform and ray cambial cells, fiber, and vessel cells [14–17]. Despite these studies, the full diversity of cell types and cell differentiation trajectories in the vascular tissues of woody plants have not yet been comprehensively analyzed, and their degree of heterogeneity is still unclear. However, advances in single-cell RNA sequencing (scRNA-seq) provide unprecedented opportunities for high-resolution characterization of gene expression [18–21]. The technique has been recently used to identify cell types in roots (*Arabidopsis* and rice), shoot apices (*Arabidopsis*, tomato and maize), leaves (*Arabidopsis* and peanut), and both ears and anthers of maize [22–36]. These studies have consistently revealed a wide range of cell heterogeneity and allowed the identification of rare and novel cell types, the characterization of multiple different cell types and states, and the establishment of detailed developmental trajectories during tissue development [20, 21]. However, the technique has not been applied in vascular tissue, especially tissues of woody plants, until its recent application in the differentiating xylem of *Populus alba* × *Populus glandulosa* [37]. This may be due to the presence of SCW with high and varying thickness, which greatly complicates isolation of individual cells without loss of their viability and purity.

In an attempt to circumvent these problems, here we applied an optimized protocol to isolate high-quality protoplasts, combined with 10x Genomic scRNA-seq technology to construct a single-cell transcriptional landscape of the poplar stem. In total, we identified 20 cell clusters covering nearly all major cell types in vascular tissue. As expected, we found a high degree of heterogeneity among these cells. In combination with in situ hybridization experiments, hundreds of cell-type-specific marker genes were identified. The differentiation trajectories of phloem and xylem cells were further reconstructed according to their transcriptome profiles. We also established a web server (https://scu-populus.shinyapps.io/scRNAPal/) to facilitate the use of our scRNA-seq data, which will provide a valuable resource for future functional studies of vascular development in trees.

## Results

### Construction of a single-cell transcriptional landscape of the poplar stem

**Fig. 1:**
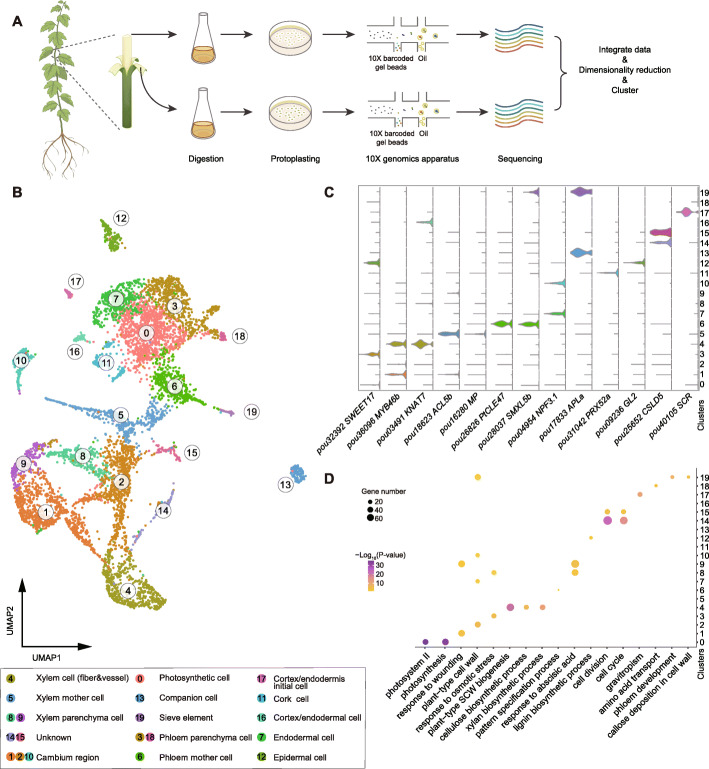
Protoplast isolation and identification of cell clusters in poplar stem. **A** Workflow of the scRNA-seq of poplar stems. Protoplasts isolated from bark and wood were loaded into a 10x Genomics Chromium Controller, separately. **B** UMAP visualization of 20 putative clusters derived from 6796 cells. Each dot denotes a single cell. Colors indicate corresponding cell clusters. **C** Violin plots showing expression of representative marker genes in each cell type. Clusters are indicated on the *y*-axis. Colors denote corresponding cell clusters. **D** Results of GO enrichment analysis of the cluster-enriched genes

To enable scRNA-seq of poplar stem cells, we harvested stems of three 4-month-old *Populus alba* var. *pyramidalis* plantlets. We then separated bark and wood tissues of parts below the third node (Fig. 1A) and obtained free protoplasts from them using a previously described digestion method [38]. The cross section of the stem showed that most of the cell types in the vascular tissue have been digested and released (Additional file 1: Fig. S1). As the isolated protoplasts were fragile and the suspension was rich in cell debris, we used an optimized method for washing and cell resuspension to maximize their purity and maintain their viability, before loading into a 10x Genomics Chromium Controller. Then, scRNA-seq libraries were constructed and sequenced using Illumina HiSeq 2000 platform. After prefiltering at both cell and gene levels, we successfully captured 3626 and 3170 cells, with median numbers of 2512 and 3330 expressed genes per cell for bark and wood tissues, respectively (Additional file 2: Table S1). In total, we detected transcripts of 27,297 and 26,653 genes in our scRNA-seq of bark and wood, respectively, covering 92.8% and 83.1% of the genes detected by bulk RNA-seq. In addition, gene expression values obtained from the combined scRNA-seq analysis showed strong correlations with the bulk RNA-seq profiles (Pearson’s *r*: 0.81 and 0.82 for bark and wood respectively, Additional file 1: Fig. S2), confirming the robustness and high quality of our scRNA-seq data. To construct a comprehensive transcriptional landscape for poplar stem, we combined these two datasets (including 6796 high-quality cells in total) for subsequent analysis.

Using the 2000 genes that showed the highest variation in expression, we scaled and reduced the combined dataset into 30 principal components (PCs) with linear dimensional reduction. The transcriptome profiles of cells were then projected in an unsupervised manner without a priori knowledge of marker genes. In total, these cells were classified into 20 clusters and visualized using *t*-distributed stochastic neighborhood embedding (t-SNE) tool and uniform manifold approximation and projection (UMAP) algorithm (Fig. 1B, Additional file 1: Fig. S3-5). Among the detected genes, 6170 specifically expressed in one or two clusters were identified as cluster-enriched (Additional file 3: Table S2, Additional file 1: Fig. S6).

### Identification of cell types with marker genes

Since very few marker genes were known for cell types of tree stems, we first compiled a list of known or predicted wood formation marker genes whose functions and expression patterns have been well studied in poplar and *Arabidopsis* (Additional file 4: Table S3, Additional file 5: Table S4), and then compared them with our identified cluster-enriched genes to annotate the clusters. Analysis of correlations between profiles derived from the scRNA-seq dataset and published high-spatial-resolution expression profiles for secondary growth of *P. tremula* [15] further validated the cell type of each cluster (Additional file 1: Fig. S7). Specifically, we found that several master regulatory genes related to xylem development including *SECONDARY WALL-ASSOCIATED NAC DOMAIN PROTEIN 1* (*SND1*), *NAC SECONDARY WALL THICKENING PROMOTING FACTOR 1* (*NST1*), and *MYB DOMAIN PROTEIN 46* and *83* [6] were highly enriched in clusters 4, 8, and 9 (Fig. 1C, Additional file 1: Fig. S8), and thus were identified as xylem cell populations. Moreover, many genes involved in SCW biosynthesis were predominantly expressed in cluster 4 (Fig. 1C, Additional file 1: Fig. S8), implying that cells in this cluster may undergo SCW thickening. These genes included the following: the xylan biosynthesis and deposition genes *IRREGULAR XYLEM 9* (*IRX9*), *IRX14-L*, and *IRX15-L* [39, 40]; SCW cellulose and hemicellulose synthase genes *COBRA-LIKE4* (*COBL4*), *CesA4*, *CesA7*, and *CesA8* [41]; genes encoding laccases (*LAC4* and *LAC17*) localized to the thick SCW of xylem vessel elements and fibers [42]; and *KNOTTED-LIKE HOMEOBOX OF ARABIDOPSIS THALIANA 7* (*KNAT7*), which negatively regulates SCW biosynthesis [43]. Furthermore, *VASCULAR RELATED NAC-DOMAIN PROTEIN 1* (*VND1*), a transcriptional regulator of SCW biosynthesis in xylem vessels [44], was also overrepresented in some cells of cluster 4, implying that vessel cells were contained in this cell cluster (Additional file 1: Fig. S8). In addition, *CINNAMYL ALCOHOL DEHYDROGENASE 7* (*CAD7*), whose expression was previously detected in xylem parenchyma cells to provide lignin precursors to the adjacent vessels and fibers [37, 45], was highly enriched in clusters 8 and 9, which were further identified as xylem parenchyma cells (Additional file 1: Fig. S8).

For phloem cells, we used *ALTERED PHLOEM DEVELOPMENT* (*APL*), a master regulator gene that promotes asymmetric cell division of phloem initials into sieve elements (SEs) and companion cells (CCs) [46], and gene members of the PHLOEM PROTEIN 2 family (*PP2-A1*, *PP2-A4*, and *PP2-A10*) that is specifically expressed in SE-CC complex [47] as markers (Additional file 6: Table S5). We found that these marker genes were highly enriched in cells of clusters 13 and 19, suggesting that they are likely phloem cells (Fig. 1C, Additional file 1: Fig. S9). In addition, we also found that *SUCROSE-PROTON SYMPORTER 2* (*SUC2*), which is essential for sucrose uptake from apoplast to CCs [48], was highly expressed in cluster 13, while several homologous genes of *SIEVE-ELEMENT-OCCLUSION-RELATED 1* (*SEOR1*), which is necessary for the formation of phloem filament in *Arabidopsis* SEs [49], were particularly enriched in cluster 19 (Additional file 1: Fig. S9). Accordingly, the cells in clusters 13 and 19 were identified as CCs and SEs respectively. Recent studies have shown that several members of the UMAMIT amino acid transporter family are specifically expressed in phloem parenchyma (PP) cells of *Arabidopsis* leaves [27]. We found that several members of this gene family (*UMAMIT9*, *UMAMIT12*, *UMAMIT20*, *UMAMIT21*, and *UMAMIT22*) were highly enriched in clusters 3 and 18 (Additional file 1: Fig. S9). Thus, cells in these two clusters were identified as PP cells. Interestingly, we found that a homolog of *SWEET17*, which is specifically expressed in vascular parenchyma cells and acts as fructose transporters in the vasculature of *Arabidopsis* leaves [50], was also highly expressed in cells of cluster 3 (Additional file 1: Fig. S9), suggesting that it may play a role in controlling the transport of fructose in the phloem of poplar.

In order to identify other bark cell populations, the regulatory module composed of *SCARECROW* (*SCR*) and *SCARECROW-LIKE 23* (*SCL23*) (Fig. 1C, Additional file 1: Fig. S10), which is required for controlling asymmetric cell division of the cortex/endodermis initial (CEI) and plays an important role in the endodermis development of *Arabidopsis* shoots and roots [51], was used as markers for the CEI cells (cluster 17). We also selected *NPF3.1* (*NRT1/PTR FAMILY 3.1*), which is specifically expressed in the endodermis [52, 53], and *CYCLIND 6;1* (*CYCD6;1*) that is involved in cortex/endodermis asymmetric stem cell division [54], as markers for endodermal cells (cluster 7) and cortex/endodermal cells (cluster 16) respectively (Fig. 1C, Additional file 1: Fig. S10). As an important component of bark, cork is characterized by the presence of both lignin and suberin (a large aliphatic biopolymer composed of long-chain fatty acids and fatty alcohols of various lengths) in the cell wall [55]. We found that the suberin biosynthetic gene, *ASFT* (*ALIPHATIC SUBERIN FERULOYL TRANSFERASE*) [56, 57], and several genes involved in biosynthesis of lignin and very long-chain fatty acids, including *PEROXIDASE 52* (*PRX52*), *PRX72*, *3-KETOACYL-COA SYNTHASE 11* (*KCS11*), *LONG-CHAIN ACYL-COA SYNTHASE 1* (*LACS1*), and *LACS2* [58, 59], were specifically expressed in cluster 11 (Additional file 1: Fig. S10). Finally, the homeobox gene *GLABRA2* (*GL2*), required for epidermal cell differentiation in *Arabidopsis* roots [60], was highly expressed in cells of cluster 12 (Additional file 1: Fig. S10), which were therefore identified as epidermal cells.

As markers for the cambium and adjacent cell populations, we used multiple genes including the key cambial regulators *WOX4* (*WUSCHEL-RELATED HOMEOBOX 4*) and *PXY* (*PHLOEM INTERCALATED WITH XYLEM*), the auxin response transcription factor *MP* (*MONOPTEROS*), and genes encoding the auxin efflux transporter *PIN1* (*PINFORMED 1*), cytokinin-responsive transcription factor gene *ANT* (*AINTEGUMENTA*), and positive regulatory peptide of cambial activity *Populus CLV3/ESR-RELATED 47* (*PttCLE47*) [61, 62]. The results support the cambium and adjacent region identities of cell clusters 1, 2, 5, 6, and 10 (Fig. 1C, Additional file 1: Fig. S11). In more detail, the three *HD-ZIP III* transcription factor genes, homologs of which (*PtrHB4* and *PtrHB7*) play critical roles in regulation of vascular cambium activity and xylem differentiation in poplars [63, 64], were highly expressed in cluster 5 (Additional file 1: Fig. S11). In contrast, *OCTOPUS* (*OPS*), a gene encoding a polarly localized plasma membrane-associated protein that plays a crucial role in protophloem formation [65], and *SMXL5* (*SUPPRESSOR OF MAX2 1-LIKE5*), which is key promoter of phloem differentiation [66], were particularly enriched in cluster 6 (Fig. 1C, Additional file 1: Fig. S11). Accordingly, we designated the cells in clusters 5 and 6 as xylem and phloem mother cells, respectively. Due to the lack of proven marker genes, we could not determine the cell types in clusters 14 and 15. However, transcriptome profiles of the cells in both clusters were highly correlated with published expression profiles of phloem and expanding-xylem tissues [15] (Additional file 1: Fig. S7), indicating that they are probably spatially distributed near the cambium. Further examination of the enriched genes revealed that genes associated with mitosis and cell cycling were strongly expressed in clusters 14 and 15, such as *CSLD5* (*CELLULOSE SYNTHASE-LIKE D5*) [67], *PLE* (*PLEIADE*) [68], *HIK* (*HINKEL*) [69], mitotic cyclin (*CYCA1;1, CYCA2;1, CYCA2;4, CYCB1;4, CYCB2;1, CYCB2;3, CYCB2;4, CYCB3;1*) [70], and cyclin-dependent kinase (*CDKB1;2, CDKB2;1*) [71] (Fig. 1C, Additional file 1: Fig. S12), indicating that this cluster is rich in dividing cells. Overall, these results confirmed the high degree of cell heterogeneity in poplar stems.

### Differentiation trajectory of phloem cells

**Fig. 2:**
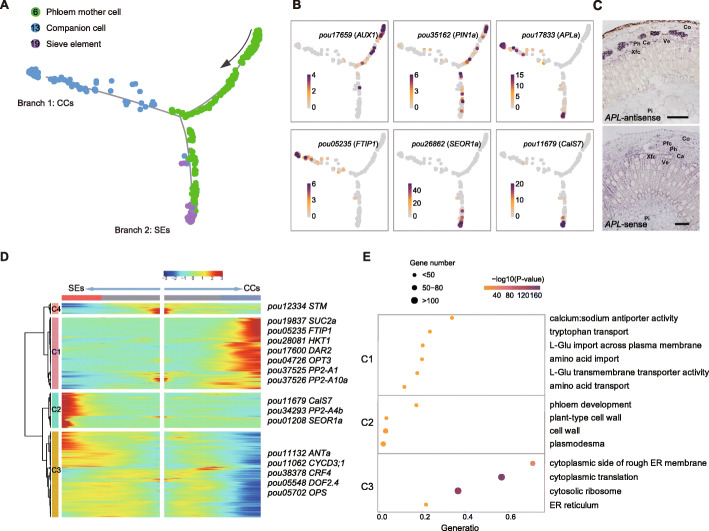
Differentiation trajectory of phloem cells. **A** A successive differentiation trajectory from phloem mother cells to SEs and CCs. Each dot indicates a single cell. The black arrow indicates the start of the trajectory. **B** Expression patterns of phloem-specific genes (*AUX1*, *PIN1*, *APL*, *FTIP1*, *SEOR1*, and *CalS7*). The colors represent expression levels of these genes in individual cells. **C** RNA in situ hybridization of *APL* with the sense probe as a negative control. Scale bars, 100 μm. **D** Heatmap showing expression of the branch-dependent genes over pseudo-time. Representative marker genes are shown on the right of the heatmap. Both sides of the heatmap are the end of pseudo-time. **E** Dot plot of GO enrichment analysis of the identified cell clusters. ER, endoplasmic reticulum. L-Glu, L-glutamate

**Fig. 3:**
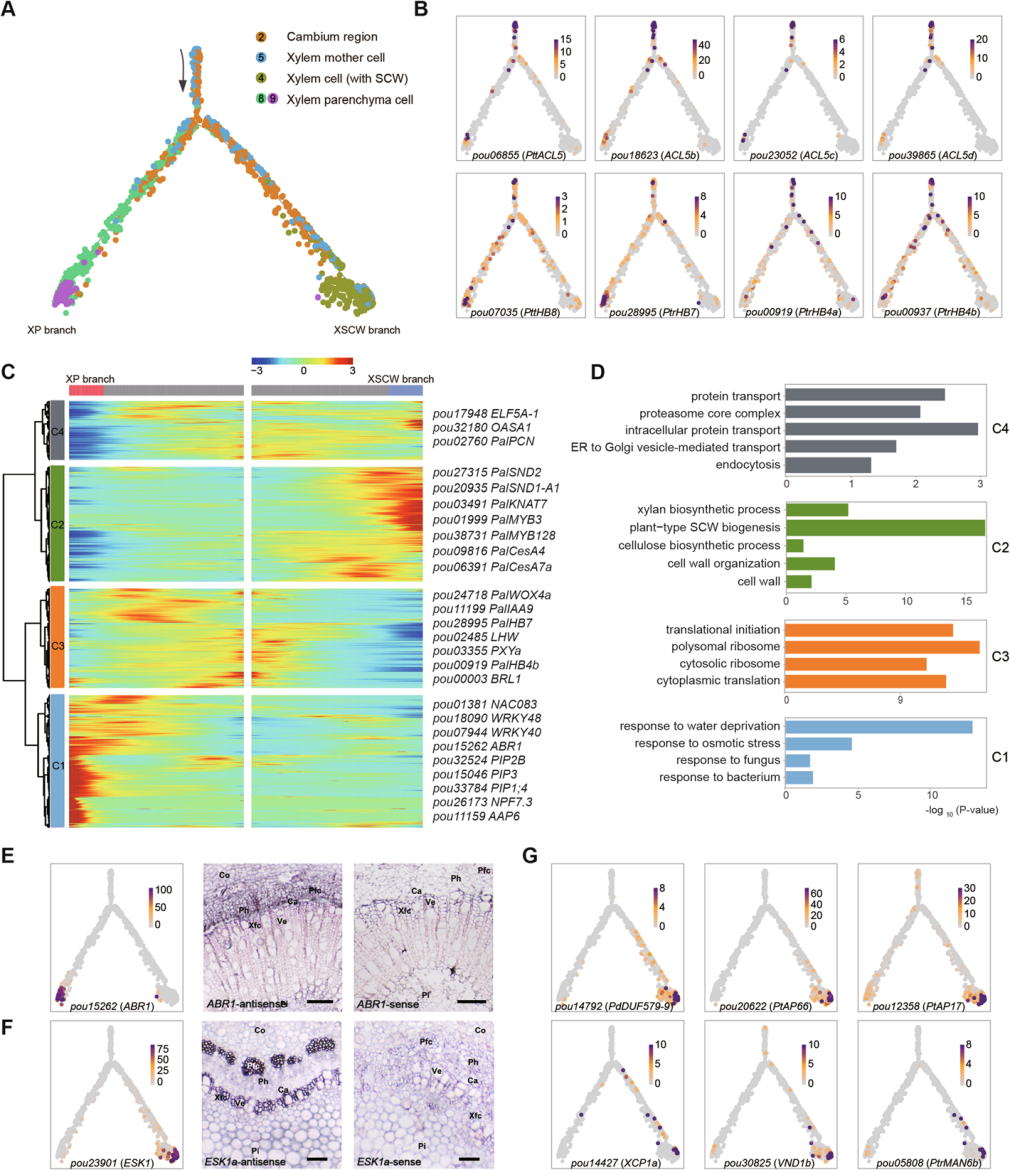
Differentiation trajectory of xylem cells. **A** The successive differentiation trajectory from xylem mother cells to mature xylem cells. Each dot indicates a single cell. The black arrow indicates the start of the trajectory. **B** Expression of representative marker genes (*ACL5*, *PtrHB4*, *PtrHB7*, and *PtrHB8*) at the beginning of the trajectory. The color bar indicates relative expression levels. **C** Heatmap showing expression levels of branch-dependent genes over pseudo-time. Representative marker genes are shown on the right of the heatmap. Both sides of the heatmap are the end of pseudo-time. **D** Bar chart showing results of GO enrichment analysis of the identified cell clusters. Expression of *ABR1* (**E**) and *ESK1a* (**F**) at the trajectory and results of RNA in situ hybridization of them with the sense probe as a negative control. Scale bars, 100 μm. **G** Representative marker genes (*PdDUF579-9*, *PtAP66*, *PtAP17*, *PtrMAN6*, *XCP1*, and *VND1*) expressed in XSCW branch. The color bar indicates relative expression levels

As the major functional domains of stems, phloem and xylem are produced through periclinal cell divisions of vascular cambium toward the outside and inside, respectively. Since the cells in intermediate and terminal developmental states were captured simultaneously by scRNA-seq, we applied Monocle2 (v2.10.0) [72] to explore the continuous differentiation trajectories of the phloem and xylem development through pseudo-time analysis (Fig. 2, Fig. 3). To construct developmental trajectory of phloem cells, phloem mother cells (cluster 6), companion cells (cluster 13), and sieve elements (cluster 19) were selected. A subset of cells from cluster 6 (phloem mother cells) assembled at the beginning of pseudo-time and gradually bifurcated into two ends representing two distinct cell clusters (Fig. 2A). We found that *SMXL5* and *OPS* were prominently expressed at the branching point and neighboring cells (Additional file 1: Fig. S13), in agreement with their key roles in phloem formation and differentiation [65, 66]. Interestingly, the auxin influx and efflux transporters *AUX1* (*AUXIN RESISTANT 1*) and *PIN1*, which are polarly localized at opposite ends of protophloem cells of *Arabidopsis* root tips [73], were also extremely highly expressed around the branching point (Fig. 2B). This result clearly suggests that they may play an important role in establishment of the auxin gradient required for regulation of cambial activity and radial growth.

In accordance with above speculation, cells from clusters 13 and 19 were located along the two branches of pseudo-time respectively, reflecting transcriptional rewiring during phloem development (Fig. 2A). More specifically, the sucrose carrier *SUC2* [48] and an essential regulator of florigen transport from phloem CCs to SEs, *FTIP1* (*FT-INTERACTING PROTEIN 1*) [74], were highly expressed in branch 1 (Fig. 2B, Additional file 1: Fig. S13), supporting the assignment of these cells as phloem CCs. In agreement with CCs’ function in providing metabolic support for SEs and promoting the loading and unloading of nutrients, genes related to “amino acid transport” were significantly enriched in this branch (Fig. 2D,E, Additional file 7: Table S6). We also found that several key strigolactone-responsive genes, including *SMXL6*, *SMXL7*, and *DWARF14* (*D14*) [75, 76], were prominently expressed in CCs (Additional file 1: Fig. S13), implying that this phytohormone and/or related signaling pathways may also be involved in differentiation of phloem cells. In contrast, the SEs marker gene *SEOR1* that is required for the formation of phloem filaments [49], and the phloem-specific callose synthase *CalS7* that is responsible for callose deposition in developing SEs [77], were enriched in branch 2 (Fig. 2B, Additional file 1: Fig. S13). The transcriptomic signature of branch 2 was initially enriched in “cytoplasmic translation,” “rough endoplasmic reticulum,” and “cytoplasmic translational elongation,” and eventually enriched in “phloem development” and “plant-type cell wall” (Fig. 2E, Additional file 7: Table S6). These observations are consistent with SEs’ differentiation process, which reportedly involves loss of ribosomes from cytoplasm, modification of endoplasmic reticulum, and cell wall thickening [78]. Interestingly, we noted that several genes homologous to *ENODL9* (*EARLY NODULIN-LIKE PROTEIN 9*), which encodes an extracellular ATP-binding protein attaching to the SE plasma membrane by a glycosylphosphatidylinositol anchor in *Arabidopsis* [79, 80], were also highly expressed in branch 2 (Additional file 1: Fig. S13), suggesting that they may play a role in SEs.

In addition, we found that many genes involved in phloem development, including the marker gene *APL* required for specific division of phloem cells [46], and *CHER1* (*CHOLINE TRANSPORTER-LIKE 1*) required for sieve plate and sieve pore development [81], were highly expressed in both branches (Fig. 2B, Additional file 1: Fig. S13). The *APL* expression was further validated by in situ hybridization assays (Fig. 2C), supporting the ordered developmental process of phloem cells. Interestingly, we found that different gene members of *PP2* gene family showed clear branch specificity in their expression patterns: *PP2-A1* and *PP2-A10* were preferentially expressed in branch 1, while *PP2-A4* was more preferentially expressed in branch 2 (Additional file 1: Fig. S13). Further molecular experiments are needed to verify their roles in the differentiation of SEs and CCs, but collectively, these results provide insights into the differentiation trajectory of phloem cells and transcriptome profiles during cell state transitions.

### Differentiation trajectory of xylem cells

Construction of the developmental trajectory of xylem, using clusters 2, 4, 5, 8, and 9, resulted in a bifurcate pseudo-time backbone representing two distinct final states (Fig. 3A), with the clusters arranged at different branch sites. As expected, most cells from clusters 2 and 5 (cambium and xylem mother cells respectively) assembled at the beginning of pseudo-time, while xylem parenchyma cells (clusters 8 and 9) and xylem cells with SCW (cluster 4) grouped into different branches, namely XP and XSCW respectively. We found that multiple *HD-ZIP III* transcription factor genes (such as *PtrHB4*, *PtrHB7*, and *PtrHB8*, all of which play crucial roles in regulation of vascular cambium activity and xylem development in poplars [63, 64, 82]) were prominently expressed at the beginning of pseudo-time (Fig. 3B, Additional file 1: Fig. S14). Similarly, four homologs of the thermospermine synthase *ACAULIS5* (*ACL5*), which forms a negative feedback loop with *HB8* that is required for proper vascular development and xylem cell specification [82], were prominently expressed around the branching point (Fig. 3B). We further investigated the differences in gene expression patterns between XP and XSCW branches (Fig. 3C). The results showed that the transcripts preferentially expressed in XP branch were initially enriched in functional categories related to “cytoplasmic translation” and eventually enriched in “response to various biotic and abiotic stresses” (Fig. 3D, Additional file 8: Table S7). Specifically, several genes encoding plasma membrane intrinsic protein (PIP) aquaporins, including *PIP1;4*, *PIP2B*, and *PIP3* were highly expressed in XP cells (Fig. 3C). In addition, we also found that multiple transcription factors associated with abiotic stress response, such as *WRKY40*, *WRKY48*, and *ABR1* (AP2-like ABA repressor 1), were highly enriched in XP cells. The expression of *ABR1* in the parenchyma cells was further validated by in situ hybridization assay (Fig. 3E). These characteristics are consistent with functions of the XP cells in carbohydrate and fat storage, water conduction, defense against pathogens, and healing and regeneration under stress conditions [83, 84].

In contrast, the XSCW branch is mainly composed of cells from cluster 4. In these cells, genes associated with “plant SCW biogenesis,” “xylan and cellulose biosynthetic process,” and “cell wall organization” were preferentially expressed (Fig. 3D), indicating that they were undergoing SCW thickening and lignification. In detail, multiple transcription factors in the core regulatory network of SCW formation, such as *SND1*, *SND2*, *KNAT7*, *MYB83*, *46*, and *103* [6], and genes encoding enzymes involved in SCW biosynthesis, such as SCW-associated cellulose synthase genes (*CesA4*, *CesA7*, and *CesA8*) [41], xylan biosynthesis genes (*IRX8*, *IRX9*, *IRX10*, *IRX14*, *IRX15*, and *IRX15-L*) [6], and lignin biosynthesis-related laccase (*LAC4* and *LAC17*) [42] were all prominently expressed in these cells (Fig. 3C, Additional file 1: Fig. S15). These genes’ expression patterns were strongly positively correlated, and binding motifs of MYB and NAC transcription factors are highly enriched in the 2-kb sequences upstream of them (Additional file 1: Fig. S16). These observations corroborate the hypothesis that members of the *NAC* and *MYB* gene families are master transcriptional regulators of SCW biosynthesis. Moreover, our results revealed high correlations in expression of homologs of *ESK1* (*ESKIMO1*), which plays an essential role in xylan acetylation during SCW biosynthesis in *Arabidopsis* [85], with these SCW-associated genes (Additional file 1: Fig. S17). The expression of *ESK1* in cells with SCW was also validated by in situ hybridization assays (Fig. 3F). Further analysis showed that multiple genes previously reported to be specifically expressed in poplar fiber cells, including *IRX15-L* (*PdDUF579-9*), *ASPARTIC PROTEASE 66* (*PtAP66*), and *PtAP17* [86–88], were particularly expressed in cells from XSCW branch (Fig. 3G). Interestingly, we found that a gene orthologous to *PtrMAN6* (*ENDO-BETA-MANNASE 6*), which is specifically expressed in poplar vessel cells [89], as well as *XCP1* (*XYLEM CYSTEINE PROTEASES 1*) and *XCP2*, both of which are reportedly vessel-specifically expressed in *Arabidopsis* [90], were also highly enriched in some cells associated with XSCW branch (Fig. 3G, Additional file 1: Fig. S15). These results imply that the XSCW branch is composed of at least two cell types, vessels and fibers. Taken together, our results clearly reveal a high degree of heterogeneity and complexity in the differentiation and fate determination of poplar xylem cells. They also confirm the robustness of our dataset and its potential utility for mining to detect new regulatory genes.

### Hormonal biosynthesis and response patterns in poplar stem cells

**Fig. 4:**
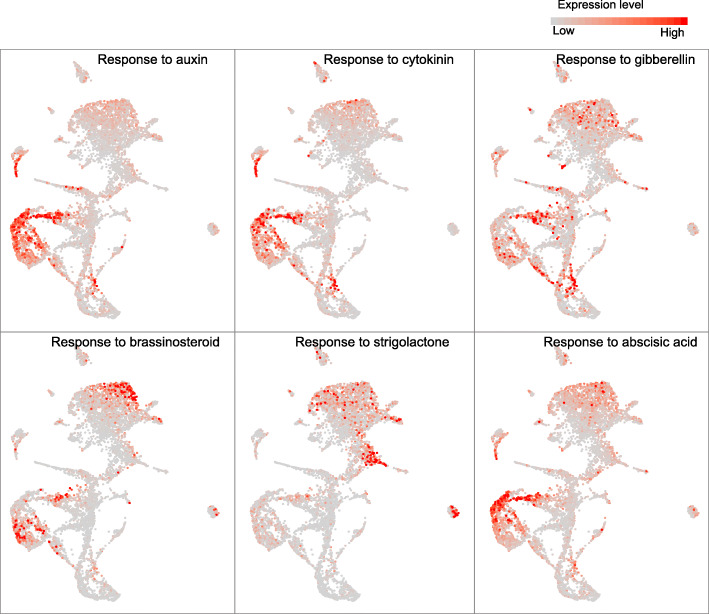
UMAP visualization of expression patterns of genes related to hormone response (auxin, cytokinin, gibberellin, brassinosteroid, strigolactone, and abscisic acid). The colors represent expression levels of these genes in individual cells

Plant hormones play key, highly interactive roles in vascular tissue development and cambium maintenance [2, 61]. By plotting the spatiotemporal expression patterns of the genes related to hormone biosynthesis and response pathways, we next analyzed the cell-specific patterns of phytohormone regulation in poplar stem. We found that biosynthesis genes of most hormones, including gibberellin acid (GA), abscisic acid (ABA), and strigolactone (SL), were mainly overrepresented in cortex and endodermal cells, while auxin, cytokinin, and brassinosteroid (BR) biosynthesis genes were also enriched in xylem cells, especially parenchyma cells (Additional file 1: Fig. S18). Specifically, all of them were not expressed in cells with SCW. Like SL biosynthesis genes, SL response genes were overrepresented in the cortex and endodermal clusters, indicating that these cells have an autocrine function (Fig. 4). However, the other hormone response genes, especially genes responsive to auxin, cytokinin and GA, were overrepresented in cambium and adjacent cells, such as cells from clusters 1 and 10. In addition, auxin-, cytokinin-, and ABA-responsive genes were also clearly overrepresented in xylem parenchyma cells (clusters 8 and 9) (Fig. 4). Collectively, these results revealed complex patterns and interactions of various hormones during poplar vascular development and enabled investigation of potential hormonal signaling at single-cell resolution.

### scRNA-seq predicts potential gene redundancy in poplar vascular development

**Fig. 5:**
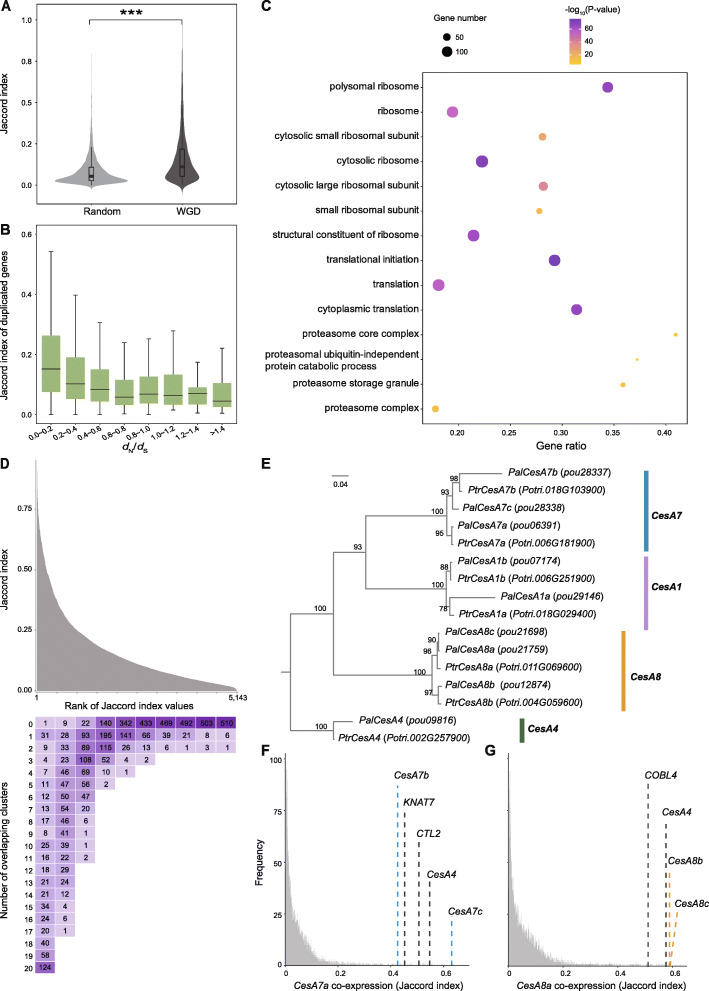
scRNA-seq predicts potential gene redundancy. **A** Comparison of Jaccard index values between WGD-derived and random selected gene pairs. Asterisks indicate highly significant differences according to the Wilcoxon signed-rank test (*P* < 0.0001). **B** Boxplot showing that Jaccard index values of correlations between WGD-derived gene pairs decreased with increases in *d*_N_/*d*_S_. **C** Dot plot of GO enrichment analysis of paralogs within the top 10% of the Jaccard index. **D** Bar chart (top) and heatmap (bottom) showing the distribution of Jaccard index values of 5143 WGD-derived paralogs, and the distribution of the number of overlapping clusters, where both copies of a gene pair were expressed in more than 25% of the cells in that cluster. **E** Phylogenetic tree of *CesA1*/*4*/*7*/*8* in *P. alba* var. *pyramidalis* and *P. trichocarpa.* **F** Frequency of Jaccard index between *CesA7a* and all other expressed genes. **G** Frequency of Jaccard index between *CesA8a* and all other expressed genes

A recent whole-genome duplication (WGD) resulted in two copies of each gene in the poplar genome [91, 92]. These paralogous genes usually exhibit functional redundancy, and knocking out one of them will not result in a clear phenotypic change, which limits functional study of these genes. To predict functional redundancy in poplar vascular development, we first identified a total of 9147 paralogous gene pairs derived from WGD in the *P. alba* var. *pyramidalis* genome. Among these, both copies of 5143 gene pairs were detected to be expressed in at least 1% of the cells captured by our scRNA-seq. In-depth analysis showed that expression patterns of these WGD-derived paralogs were significantly more strongly correlated (higher Jaccard co-expression index values) than the genomic background (*P* value < 0.0001, Wilcoxon signed-rank test), and the correlation declined with increasing protein sequence differentiation (Fig. 5A,B), indicating that the functional redundancy between these paralogs is modulated by protein sequence evolution and expression pattern differentiation. Functional enrichment analysis revealed that the paralogs falling within the top 10% of the Jaccard index distribution were associated with basic cellular component and maintenance, such as “small/large ribosomal subunit,” “cytosolic ribosome,” “proteasome complex,” and “translation initiation” (Fig. 5C, Additional file 9: Table S8, Additional file 10: Table S9), which is consistent with the observation that the duplicated genes related to basic cellular machinery in *Arabidopsis* have a slower divergence rate at both sequence and expression levels [93].

In order to further investigate functional redundancy of the paralogs across cell clusters, we defined that if both copies of a gene pair were expressed in more than 25% of the cells in a certain cluster, they were considered to be overlapping in that cluster. Approximately 56.8% (2921) of the paralogs did not show any overlapping clusters, which is consistent with their low expression correlation (Fig. 5D). However, we found that many paralogs with overlapping clusters were also detected as cluster-enriched genes, especially in clusters 4, 9, 14, and 15 (Additional file 1: Fig. S19), indicating that functional redundancy may be more frequent in these cell types. Consistent with the basic cellular functions of the paralogs in the top 10% of the Jaccard index, most of them exhibited overlapping expression patterns in almost all cell clusters (Fig. 5D). Despite this, we identified 40 gene pairs with the highest expression similarity but showing only one or two overlapping clusters, strongly suggesting that their functions are redundant in these cell types (Additional file 9: Table S8). Interestingly, we noted that most of these paralogs were specifically expressed in xylem cells with SCW (cluster 4), including *IQD10*, *MYB46*, *SND2*, *CesA7*, and *CesA8*. Previous studies have shown that both *CesA7* (*PtrCesA7A* and *7B*) and *CesA8* (*PtrCes8A* and *8B*) gene pairs in the *P. trichocarpa* genome are functionally redundant, so the *ptrcesa7a/b* or *ptrcesa8a/b* double mutants, but not single mutants, have lower cellulose contents than wild-type counterparts and abnormal morphology [94, 95]. In the *P. alba* var. *pyramidalis* genome, we detected three copies of both *CesA7* and *CesA8* (Fig. 5E). In addition to the paralogs derived from the WGD, there is another copy of both of these genes due to independent gene duplication events in this species. Our data revealed strong co-expression of the three copies of *CesA7* and three copies of *CesA8* (Fig. 5F,G), supporting that functional redundancy in poplar vascular development could be predicted by co-expression networks in scRNA-seq. Overall, these results highlight the utility of poplar stem scRNA-seq data for predicting gene redundancy.

## Discussion

scRNA-seq is a revolutionary technology with far greater power to identify new cell types and reveal cellular heterogeneity at high resolution than bulk RNA-seq [18, 19]. Recently, it has been widely used to elucidate cell differentiation and development processes of herbs or crops, including *Arabidopsis*, rice, maize, and tomato, particularly in young and/or easily dissociated tissues, such as root tips, shoot apices, and leaves [23, 26, 27]. However, similar studies on tree species or highly lignified plant tissues are still challenging, due to the presence of thick SCW that is difficult to digest to release single cells. However, the optimized protocol applied in this study enabled protoplast isolation and subsequent generation of a comprehensive single-cell transcriptional landscape of the poplar stem. We identified numerous cell clusters and state changes associated with vascular development, including xylem, phloem, cambium, epidermis, and endodermis cells (Fig. 1B). These results confirmed that our method is sensitive enough to detect most poplar genes and suggest that our optimized or further improved protocol can be potentially applied to more tree species or lignified plant tissues.

Nevertheless, it should be noted that processes involved in cell fate determination and pattern formation in poplar stems are still poorly understood, and some cell types were defined by marker genes that are either homologs of *Arabidopsis* genes or genes associated with related biological processes. Moreover, the cells in poplar vascular tissue are surrounded by cell walls with large variations in thickness and components, making it difficult to both obtain sufficient single cells and avoid changes in gene expression during protoplast isolation. For example, we have not detected a cell type or state in which genes related to programmed cell death are strongly expressed. This may be due to the thickened SCW of these cells hindering efficient isolation of protoplasts from them. Similarly, we found that only a few cork cells (cluster 11) were captured by our scRNA-seq (Additional file 1: Fig. S1), which may be due to the presence of suberin in their cell wall that can strongly affect the release of protoplasts [96]. In addition, although we have excluded the effect of the cell-cycle genes before cell clustering, we still found that the genes involved in cell cycling and division were specifically expressed in clusters 14 and 15, without any other confirmed marker genes to determine their cell types (Fig. 1D, Additional file 1: Fig. S12). Therefore, further improvement of the protocol for preparing protoplasts from these cell types is needed, and future results of scRNA-seq analysis must be carefully verified using other techniques, such as promoter reporter analysis and cell lineage tracing. Alternatively, application of single nucleus RNA sequencing and/or spatial transcriptomics technologies to vascular tissue may address these issues [97, 98].

Our results clearly confirm the power of scRNA-seq to reveal cellular heterogeneity, discover new cell types, and characterize cell states along the developmental trajectory of poplar stems that would be missed by lower resolution sequencing. For example, our scRNA-seq data revealed two subtypes of xylem parenchyma cells and their state transitions along the differentiation trajectory of xylem cells (Fig. 3). Our results also revealed that the cells within and/or around the vascular cambium are highly heterogeneous, although we cannot faithfully construct a continuous differentiation trajectory of these cells, possibly due to the relatively low representation of procambium and precursor cells, or failure of our current protocol to capture some intermediate cell types. Nevertheless, the identified multiple cell-type-specific marker genes (Additional file 6: Table S5), together with the gene expression dynamics along the developmental trajectories of phloem and xylem cells, provide insights with unprecedented resolution into vascular cell differentiation in poplar stems. Finally, co-expression networks derived from scRNA-seq data such as the set obtained in this study have clear utility for identifying new regulatory genes and predicting the functional redundancy of homologous genes. In the future, molecular functional verification of these candidate markers will help to understand the cell differentiation and fate determination during the vascular development of trees at single-cell resolution.

## Conclusions

In this study, we applied scRNA-seq technology to highly lignified poplar stems and found that they are composed of highly heterogeneous cells. We identified a total of 20 putative cell clusters with a series of novel cell-type-specific marker genes, whose expression dynamics were further used to reconstruct the cell differentiation trajectories involved in phloem and xylem development. We also investigated the complex hormonal response patterns of these cell types during poplar vascular development, and emphasized the use of our data to predict potential gene redundancy and identify candidate genes related to vascular development in trees. In conclusion, our research established a transcriptional landscape of the major cell types of poplar stems at single-cell resolution and provided a valuable resource for studying the basic principles of vascular cell specification and differentiation in trees.

## Methods

### Poplar growth conditions and protoplast isolation

*Populus alba* var. *pyramidalis* plantlets were grown in a greenhouse with a photoperiod of 16 h light/8 h darkness and 60% humidity. The stems below the third internode of three 4-month-old poplars were harvested, separated into bark and wood, and cut into 1-cm segments. The segments from bark and wood tissues were mixed separately, and then respectively submerged in cell wall digestion solution consisting of 1.5% (wt/vol) cellulase R-10, 0.4% (wt/vol) macerozyme R-10, 0.5 M mannitol, 20 mM KCl, 10 mM CaCl_2_, and 0.1% (wt/vol) bovine serum albumin [38]. And the mixtures were placed in a shaker at 50–55 rpm for 2.5 h in the dark at room temperature to release protoplasts respectively. The protoplasts were filtered with a 40-μm cell strainer to eliminate any clumped cells, and collected by centrifugation at 100×*g* for 4 min. Since the protoplasts were very fragile in the washing buffers recommended by 10x Genomics, we used W5 solution (5 mM glucose, 2 mM MES (pH 5.7), 154 mM NaCl, 125 mM CaCl_2_ and 5 mM KCl) to gently wash and resuspend the protoplasts three times. After removing the supernatant, the protoplasts were resuspended by adding a small amount of digestion solution without enzyme to avoid the influence of excessive calcium on the reverse transcription reaction. The concentration and viability of the protoplasts were estimated by trypan blue staining, and high-quality protoplasts with viability ≥ 75% were immediately processed with the 10x Genomics Single Cell Protocol (CG00052, RevC).

### scRNA-seq library construction and sequencing

The scRNA-seq libraries were constructed using the 10x Chromium Single Cell 3′ Platform according to the manufacturer’s instructions (10x Genomics). Briefly, the protoplasts isolated from bark or wood tissues were loaded into a Chromium microfluidic chip with 3′ v2 chemistry and barcoded with a 10x Chromium Controller to generate single cell GEMs (gel beads in emulsion). scRNA-seq libraries were subsequently prepared using a Chromium Single Cell 3′ reagent kit v2 (10x Genomics, Pleasanton, CA). The DNA libraries were qualitatively analyzed using an Agilent 2100 Bioanalyzer, and paired-end reads were produced by an Illumina HiSeq 2000 platform.

### scRNA-seq data pre-processing

The raw scRNA-seq reads from wood and bark tissues were separately aligned to the *P. alba* var. *pyramidalis* reference genome [99], using the Cell Ranger pipelines (https://support.10xgenomics.com/single-cell-gene-expression/software/pipelines/latest/what-is-cell-ranger) with default parameters (v2.0, 10x Genomics), and expression matrices for each gene and each cell were generated. The gene-cell matrices were then loaded into the Seurat package (v3.1.0) for further analysis [100]. To remove the low-quality genes and cells, only the genes that were expressed in at least three cells were considered, and we filtered the cells with expressed genes over 9000 or fewer than 200. Cells with less than 500 or more than 70,000 unique molecular identifiers (UMIs) were also filtered out, as well as cells with more than 5% mitochondrial genes. In addition, doublets (two or multiple cells in one oil droplet) in each scRNA-seq dataset were detected with DoubletFinder (v2.0.3) [101] using the number of artificial doublets (pN) of 0.25. To identify the optimal neighborhood size (pK), the function “paramSweep_v3” was executed using parameter “PCs = 1:20” and the maximum of pK value was selected as an optimal pK parameter. For the estimate of the number of expected real doublets (nExp), the doublet formation rate was assumed as 7.5% and nExp value was then adjusted according to homotypic doublet proportion. Finally, the parameters of nExp, pN, and pK were set to “223, 0.25, 0.005” and “265, 0.25, 0.3” for wood and bark dataset respectively, and only cells marked with “Singlets” were retained for further analysis. After filtering, 3626 and 3170 cells from bark and wood tissues, respectively, remained in the matrix. We integrated these two scRNA-seq datasets into an experiment-wide feature-barcode matrix for subsequent analysis, using combined canonical correlation analysis (CCA) [102] and mutual nearest neighbors (MNN) [103] methods in the Seurat package (v3.1.0) [100].

### Cell clustering and identification of cluster-enriched genes

The scaled gene-cell matrix obtained as described above was normalized by “LogNormalize” in Seurat (v3.1.0) [100]. To mitigate the effects of protoplasting and cell-cycle heterogeneity on cell clustering [26], the cell cycle score and the proportion of protoplasting genes in each cell were calculated by the “CellCycleScoring” and “PercentageFeatureSet” function provided in Seurat. The cell-cycle and protoplasting genes were identified as the closest homologous genes to *Arabidopsis*, which are provided by two published papers [104, 105]. The variations resulted from cell-cycle and protoplasting genes were regressed out by the “ScaleData” function using “vars.to.regress.” Then, we identified the highly variable genes using “FindVariableFeatures” with “selection.method = "vst"” parameter, and the top 2000 genes were used for linear dimensionality reduction by principal component analysis (PCA). The first 30 PCs were used as inputs for a graph-based approach to identify cell clusters [106], which were further visualized and explored by *t*-SNE (with parameter “dims = 20”) [107] and UMAP (with parameter “umap.method = "umap-learn"”) [108]. We also identified cluster-enriched genes using “FindAllMarkers” with parameters “min.pct = 0.25” and “logfc.threshold = 0.58” implemented in Seurat (v3.1.0) [100]. Cluster-specific marker genes were finally selected from these cluster-enriched genes that were expressed in at least 25% of the cells in a cluster, but less than 25% of the cells in all other clusters.

### Sequencing and analysis of bulk RNA-seq

The stems below the third internode of a 4-month-old poplar were harvested, separated into bark and wood, and frozen immediately for bulk RNA sequencing. Briefly, total RNA extracted from each tissue was used for library construction and deep sequencing by BGISEQ-500 (BGI-Shenzhen, China). The obtained clean reads were aligned to the reference genome [99] using HISAT2 (v2.1.0) [109], and then the expression levels were calculated and normalized by StringTie (v1.3.3b) [110]. Finally, the Pearson correlations between bulk RNA-seq and scRNA-seq datasets were calculated in R. In addition, the transcriptome data of cryosections from the secondary phloem, vascular cambium, and wood-forming tissues of *P. tremula* were downloaded [15]. MCScanX [111] software was used to identify 17,403 orthologs between *P. tremula* and *P. alba* var. *pyramidalis*. Then Pearson correlations between gene expression levels of our scRNA-seq and the RNA-seq of cryosections were calculated.

### RNA in situ hybridization

We conducted mRNA in situ hybridization as previously described [112]. Briefly, fifth internode of 4-month-old poplar were collected and immediately fixed in FAA (10% formaldehyde, 5% acetic acid, 47.5% ethanol) for 48 h. The fixed tissue was dehydrated through a graded alcohol series (70, 85, 90, 95, and 100%) and a HistoClear series, then embedded in paraplast. Then, 10-μm sections were cut and mounted on Pro-beOn Plus Slides (Fisher Scientific, 22-230-900). *APL*, *ABR1*, and *ESK1a* probes were labeled using a DIG RNA Labeling Kit (Roche). Primer sequences for all genes were listed in Additional file 11: Table S10. And then hybridization and detection of hybridization signals were performed as previously described [112] using a DIG Nucleic Acid Detection Kit (Roche).

### Pseudo-time analysis

To explore the developmental trajectories of specific cell clusters, the R package Monocle2 (v2.10.0) [72] was used. Briefly, the subset of raw data from target cell clusters were first extracted, and the variance in each gene’s expression across cells was calculated using “dispersionTable.” Variable genes were then determined based on average expression level (with parameters “mean_expression > = 0.1” and “dispersion_empirical > = 1 * dispersion_fit”) to define a developmental progress. The dimensionality of the data was subsequently reduced to two components using the “DDRTree” method, and the trajectory was inferred with the “orderCells” function. The pseudo-time trajectory was then plotted using the “plot_cell_trajectory” function in Monocle2 (v2.10.0) [72]. Finally, we used “BEAM” to identify the branch-dependent or pseudo-time-dependent genes, which were visualized by the “plot_genes_branched_heatmap” function. The dynamical expression of genes along the pseudo-time were visualized by the “plot_pseudotime_heatmap” function in Monocle2 (v2.10.0) [72].

### Functional and motif enrichment analysis

We performed Gene Ontology (GO) enrichment analysis to detect and evaluate cluster-specific or branch-dependent genes with an in-house perl script (https://github.com/wk8910/bio_tools/blob/master/15.GO_analysis/02.go_enrichment/). For GO enrichment, all genes, bark-expressed genes, and wood-expressed genes were selected as background sets in cluster-specific (Fig. 1D), phloem-branch-dependent (Fig. 2E), and xylem-branch-dependent (Fig. 3D) analysis, respectively. *P* value was obtained by Fisher’s exact test and adjusted by Benjamini-Hochberg false discovery rate. Motif enrichment analysis of interesting gene sets was performed using a built-in script of Homer (findmotif.pl) [113], with promoters of wood-expressed genes as the background and “*Arabidopsis*” as “Promoter Sets.”

### scRNA-seq co-expression analysis

To perform co-expression analysis using our scRNA-seq data, Pearson’s correlation coefficient between the expression level of each gene pair was calculated to identify genes with similar expression patterns. And the gene correlation network was displayed using Cytoscape (v3.7.1) [114]. In addition, we calculated Jaccard index value (also known as Jaccard similarity coefficient) for each pair of genes expressed in at least 1% of the captured cells [28]. For each gene, the cells with at least 1 UMI were assigned a value of 1, and all other cells were assigned a value of 0. To explore functional redundancy between paralogs, we first identified WGD-derived paralogs as previously described [99], and filtered the gene pairs if one or both copies lacked UMI values. In total, 5143 WGD-derived paralogs were finally retained to compare their Jaccard index values. The non-synonymous (*d*_N_) and synonymous (*d*_S_) substitution rate and their ratio were estimated using the “yn00” function in PAML (v4.9e) [115].

#### Review history

The review history is available as Additional file 12.

### Supplementary Information

**Additional file 1: Fig. S1-S19.** Supplementary figure legends and supplementary figures (Fig. S1-S19).

**Additional file 2: Table S1.** Summary statistics of scRNA-seq data for bark and wood tissues.

**Additional file 3: Table S2.** List of the cluster-enriched genes.

**Additional file 4: Table S3.** List of genes that have been functionally studied in poplars.

**Additional file 5: Table S4.** Gene list used for cell-type identification in this study.

**Additional file 6: Table S5.** Cluster-specific marker genes.

**Additional file 7: Table S6.** Results of GO enrichment analysis for genes in Fig. 2D.

**Additional file 8: Table S7.** Results of GO enrichment analysis for gene clusters in Fig. 3C.

**Additional file 9: Table S8.** Information of the WGD-derived gene pairs falling within the top 10% of the Jaccard index distribution.

**Additional file 10: Table S9.** GO enrichment analysis for paralogs falling within the top10% of the Jaccard index distribution.

**Additional file 11: Table S10.** Primers used for RNA in situ hybridization in this study.

**Additional file 12.** Review history.

## Data Availability

All sequencing data generated in this study have been submitted to the National Genomics Data Center (NGDC; https://bigd.big.ac.cn/bioproject) under BioProject accession number PRJCA005543 [116]. A supplementary online web server (https://scu-populus.shinyapps.io/scRNAPal/) was also developed to facilitate the use of our datasets.
