# Supplementary material for: Transcriptional landscape of highly lignified poplar stems at single-cell resolution
Source: Genome Biol. 2021 Nov 22;22:319. doi: 10.1186/s13059-021-02537-2 (PMC8607660; doi:10.1186/s13059-021-02537-2)
Supplement: Supplementary file 1 — Additional file 1: Fig. S1-S19. Supplementary figure legends and supplementary figures (Fig. S1-S19). [file 13059_2021_2537_MOESM1_ESM.pdf]

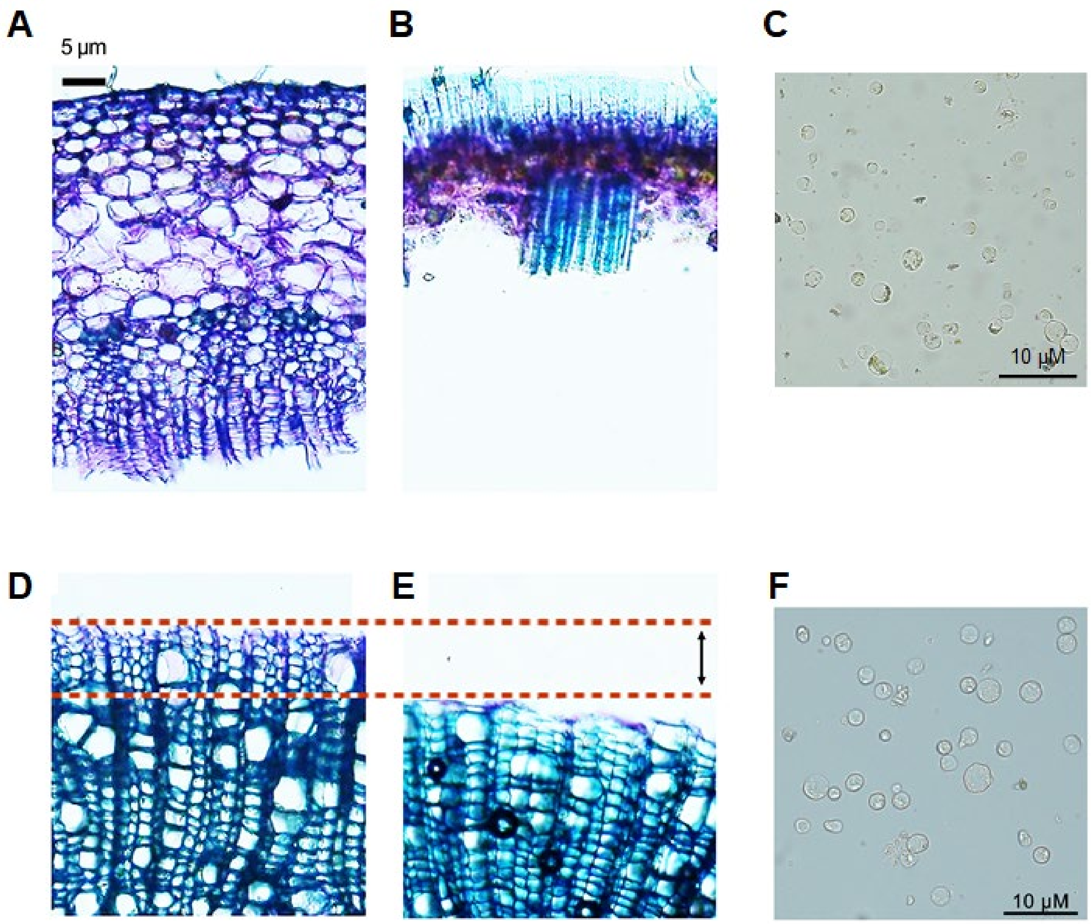

Fig. S1. Protoplast isolation of bark (A-C) and wood (D-F) tissues of poplar stem. A cross section of the bark side showing the tissue before (**A**) and after (**B**) digestion, and the protoplasts isolated from bark tissue (**C**). A cross section of the wood side showing the tissue before (**D**) and after (**E**) digestion, and the protoplasts isolated from woody tissue (**F**).

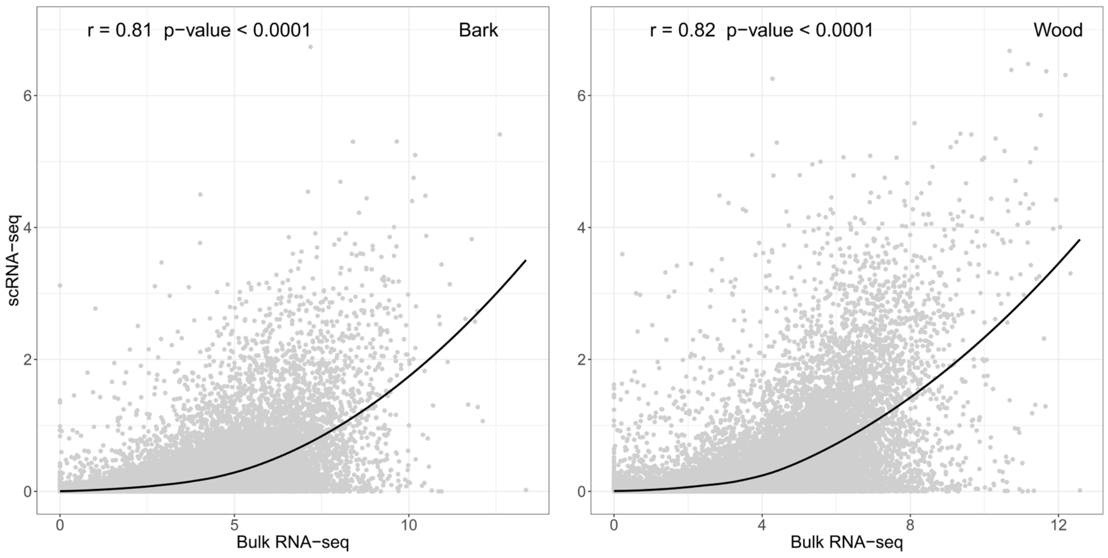

Fig. S2. Correlation analysis of gene expression between bulk RNA-seq and scRNA-seq profiling (Spearman, fitline by LOESS). The gene expression levels of scRNA-seq were inferred by the average of the expression in all cells.

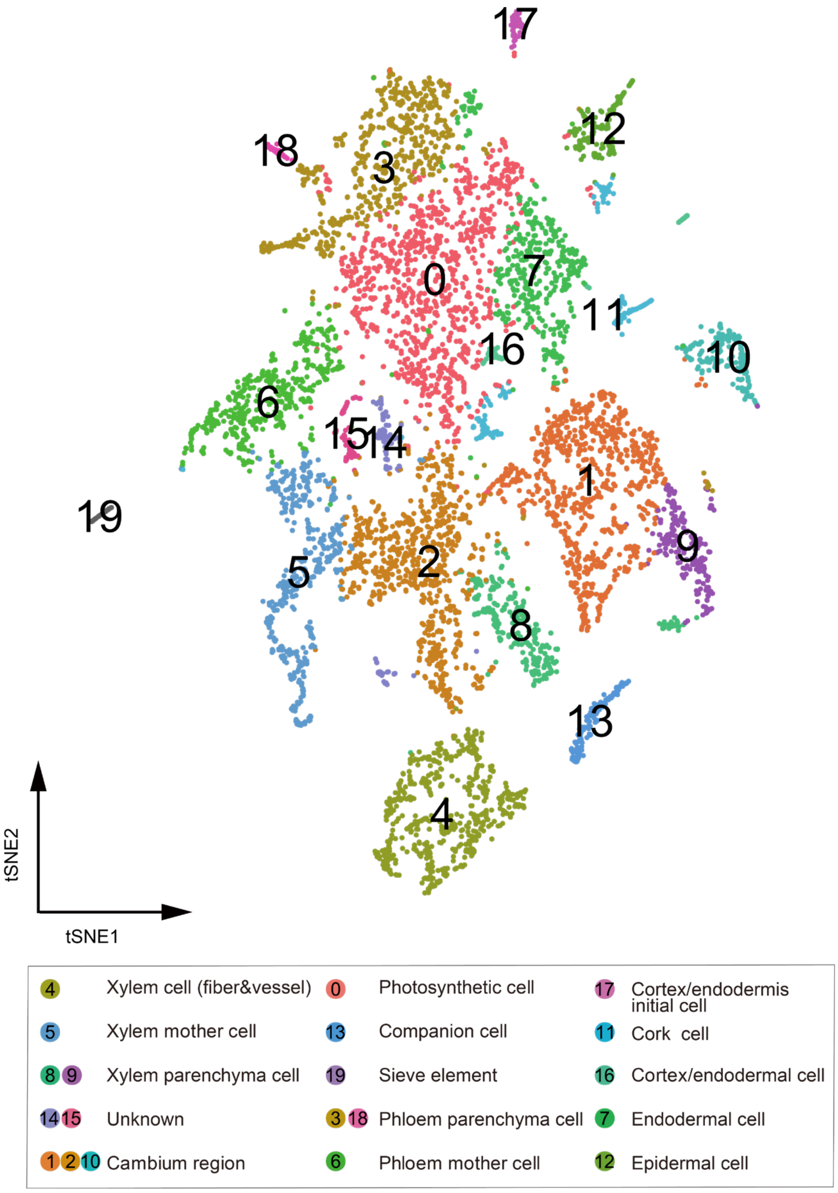

Fig. S3. Visualization of the 20 clusters of 6,796 cells by the t-SNE algorithm. Each dot represents a single cell. Colors denote corresponding cell clusters.

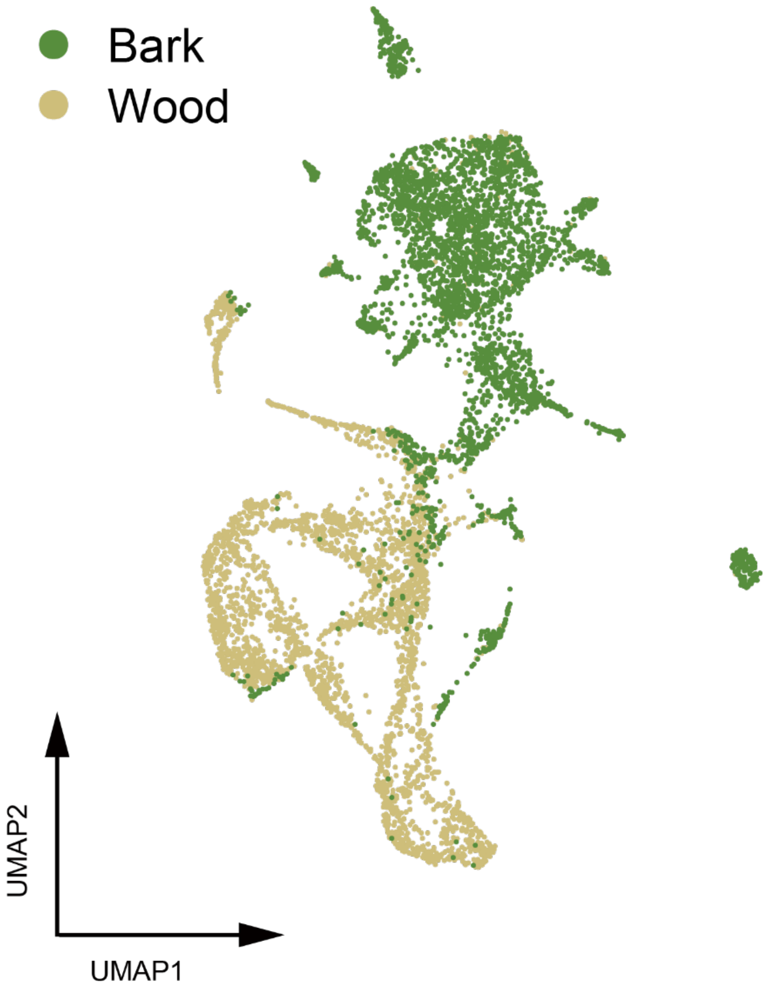

Fig. S4. Visualization of the 3,626 and 3,170 cells from bark (green) and woody (yellow) tissues, respectively, by the UMAP algorithm.

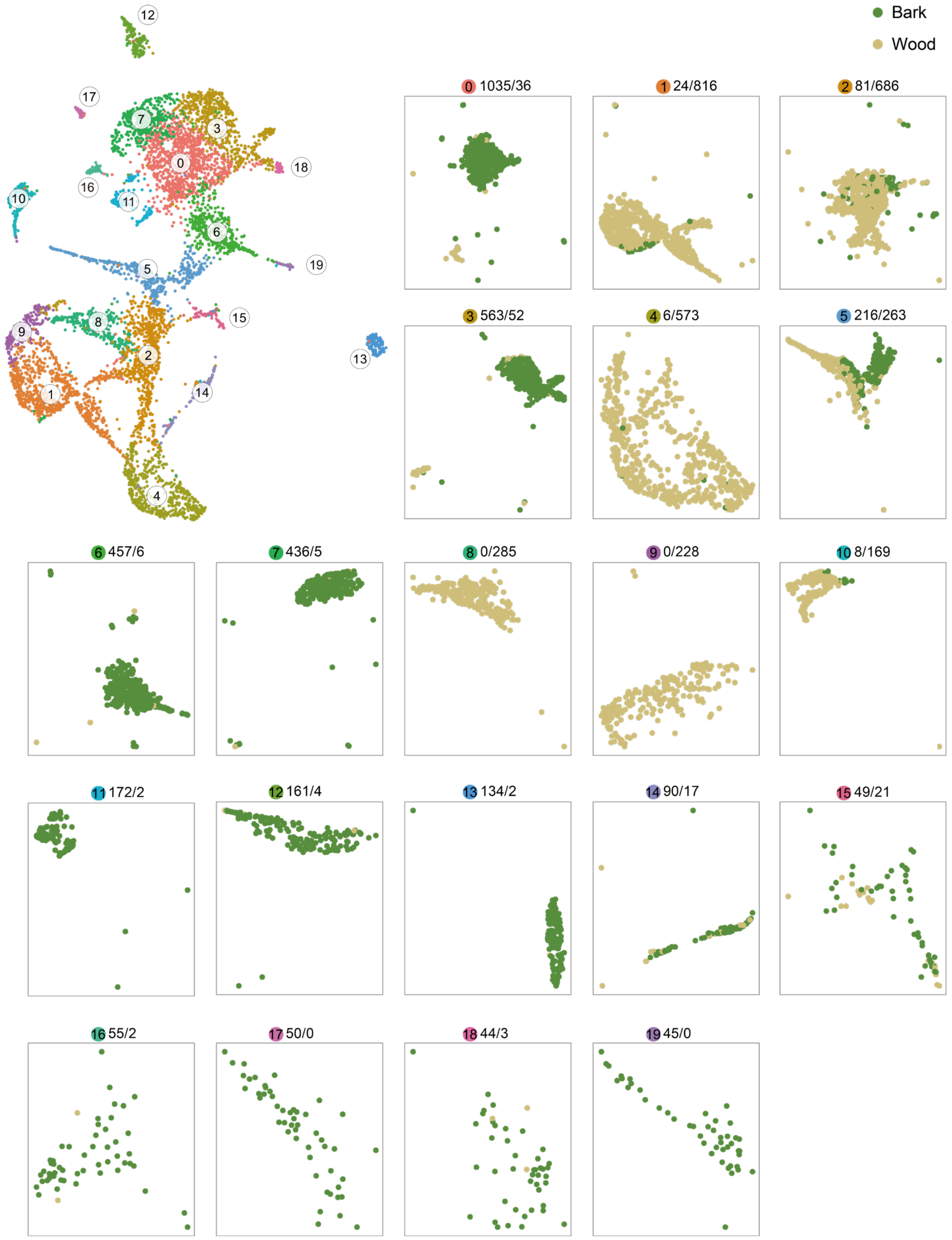

Fig. S5. UMAP visualization of the 20 cell clusters, colored by green and yellow for bark and woody tissue respectively. Each dot denotes a single cell. Numbers above each cluster represent the cell number from bark and woody tissue respectively.

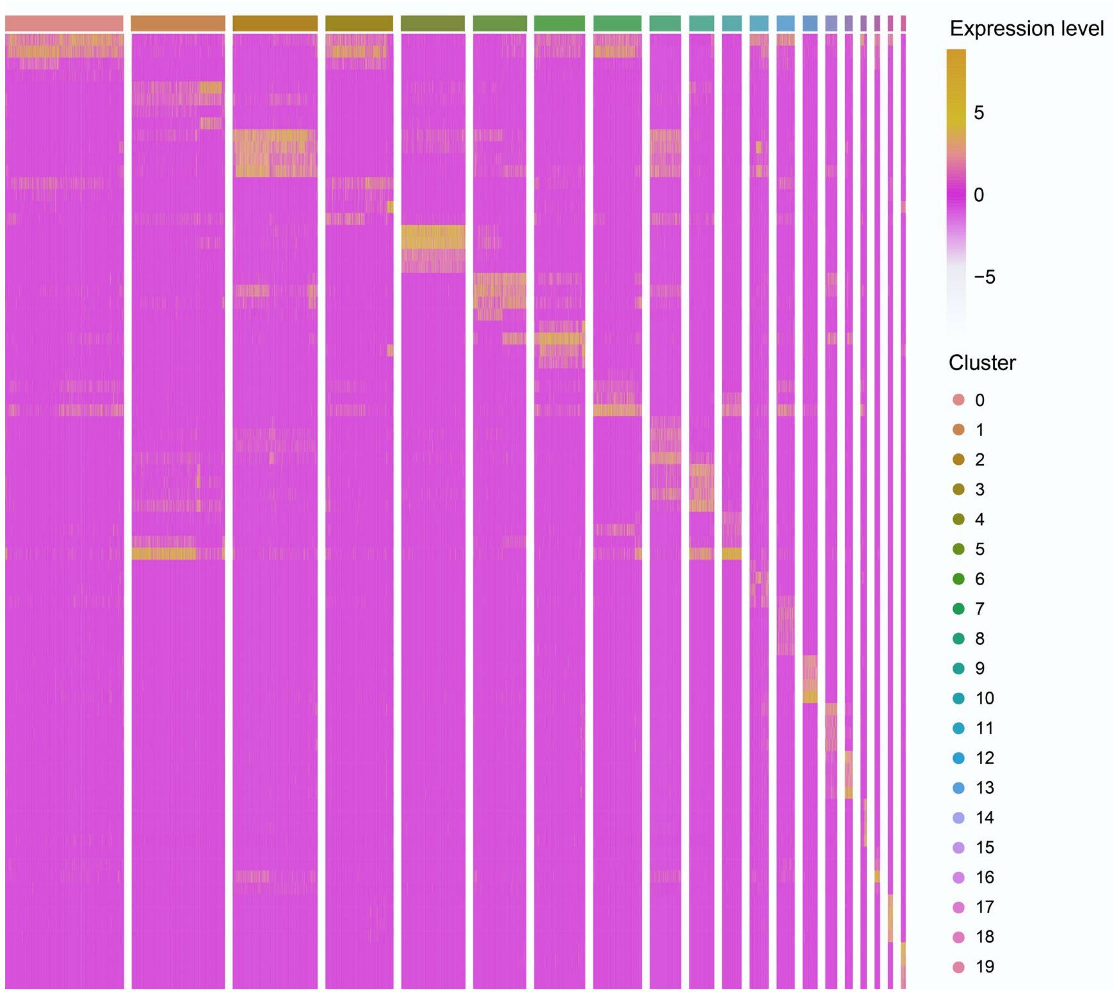

Fig. S6. Heatmap of top 4 enriched genes for each cell cluster. The color bar indicates the relative expression level.

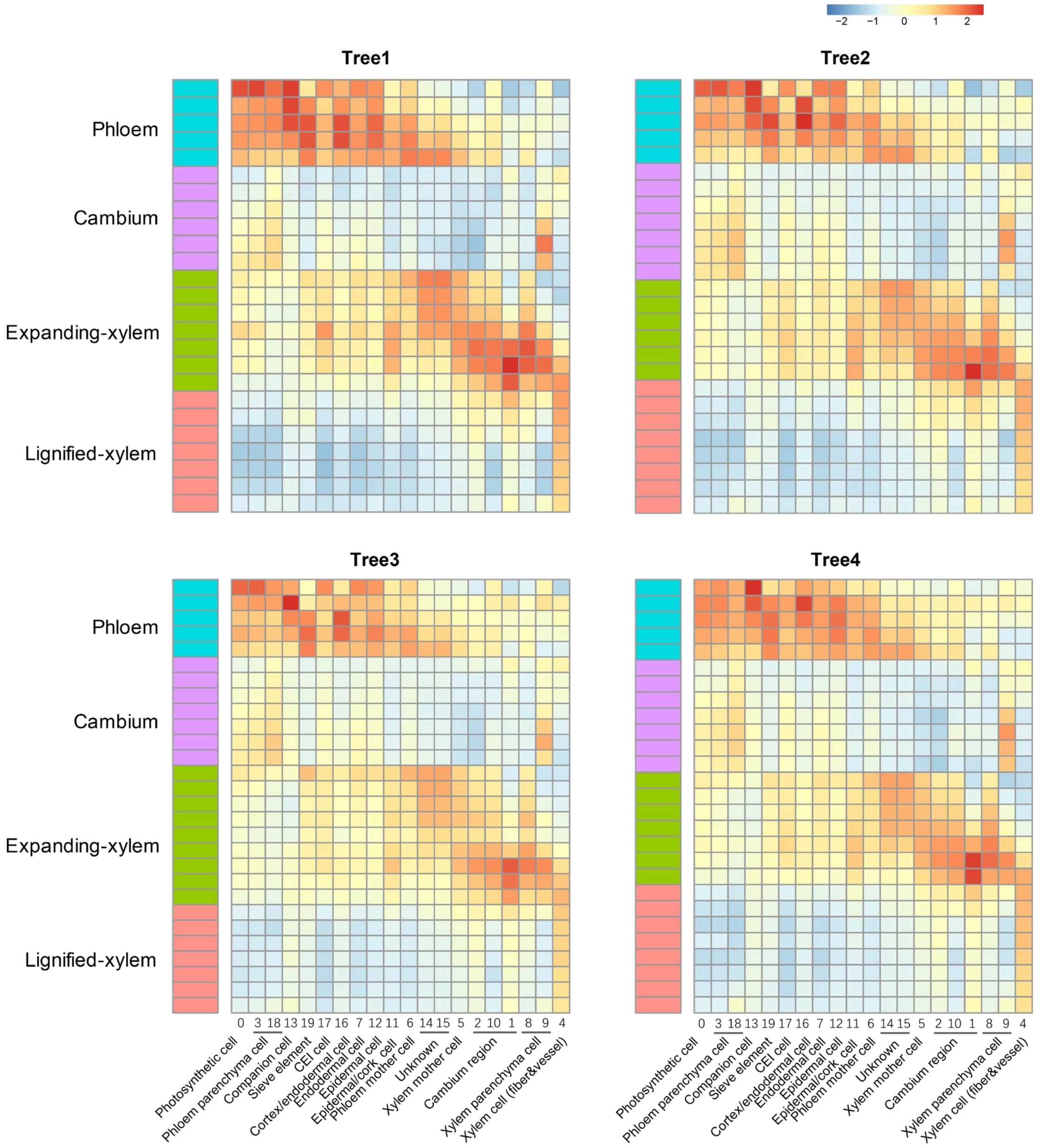

Fig. S7. Heatmap showing normalized coefficients of Pearson correlation between the 20 cell clusters identified in our scRNA-seq analysis (x-axis) and transcriptomes of cryosections (y-axis) across wood-forming tissues in four aspen clones.

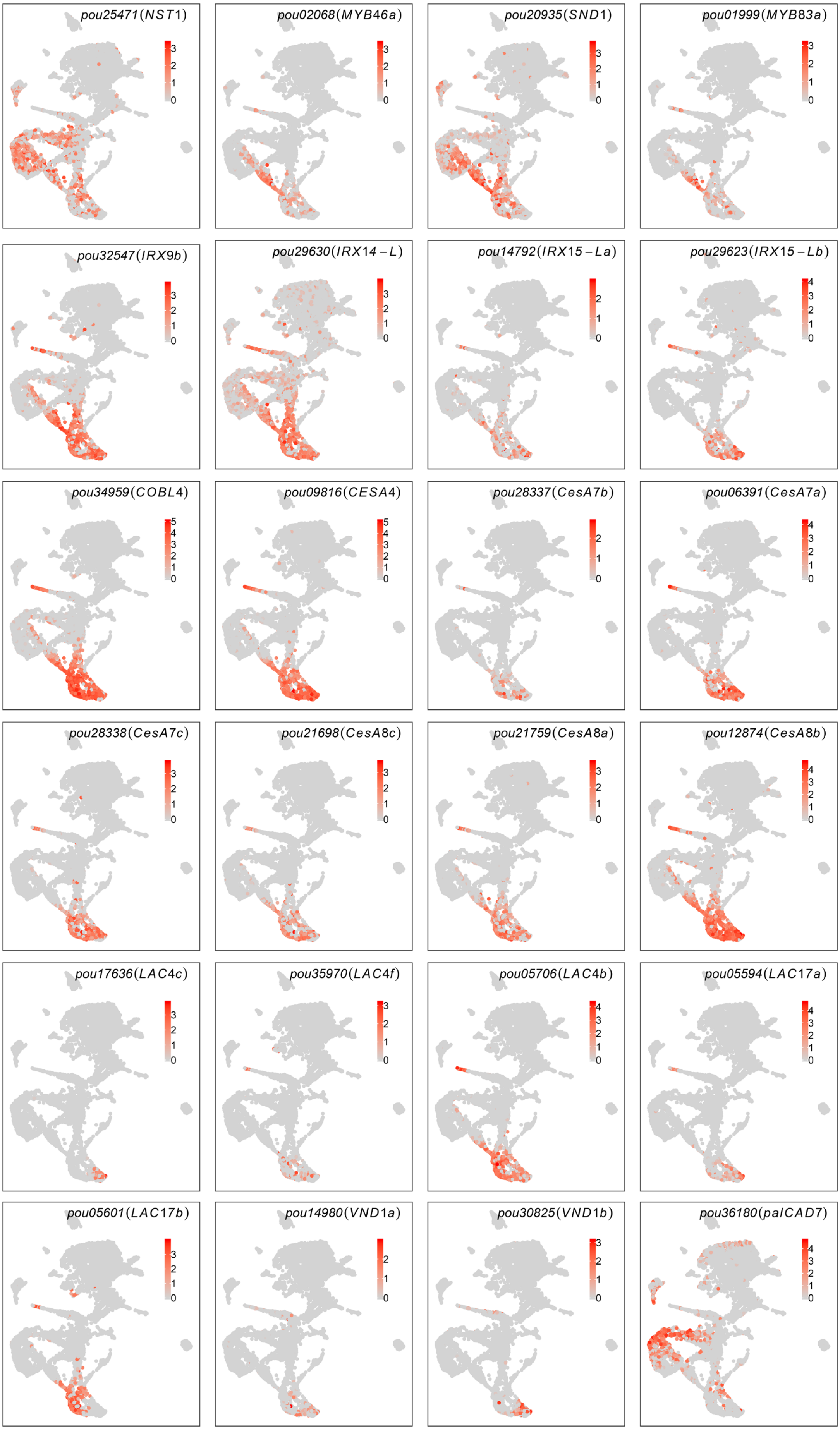
pou25471(NST1)
pou02068(MYB46a)
pou20935(SND1)
pou01999(MYB83a)
pou32547(IRX9b)
pou29630(IRX14 – L)
pou14792(IRX15 – La)
pou29623(IRX15 – Lb)
pou34959(COBL4)
pou09816(CESA4)
pou28337(CesA7b)
pou06391(CesA7a)
pou28338(CesA7c)
pou21698(CesA8c)
pou21759(CesA8a)
pou12874(CesA8b)
pou17636(LAC4c)
pou35970(LAC4f)
pou05706(LAC4b)
pou05594(LAC17a)
pou05601(LAC17b)
pou14980(VND1a)
pou30825(VND1b)
pou36180(palCAD7)

Fig. S8. Expression patterns of representative xylem-specific marker genes. The names in parentheses indicate the closest homologous genes in *Arabidopsis*, and lowercase letters indicate the numbers of multiple gene copies in *Populus alba* var. *pyramidalis*.

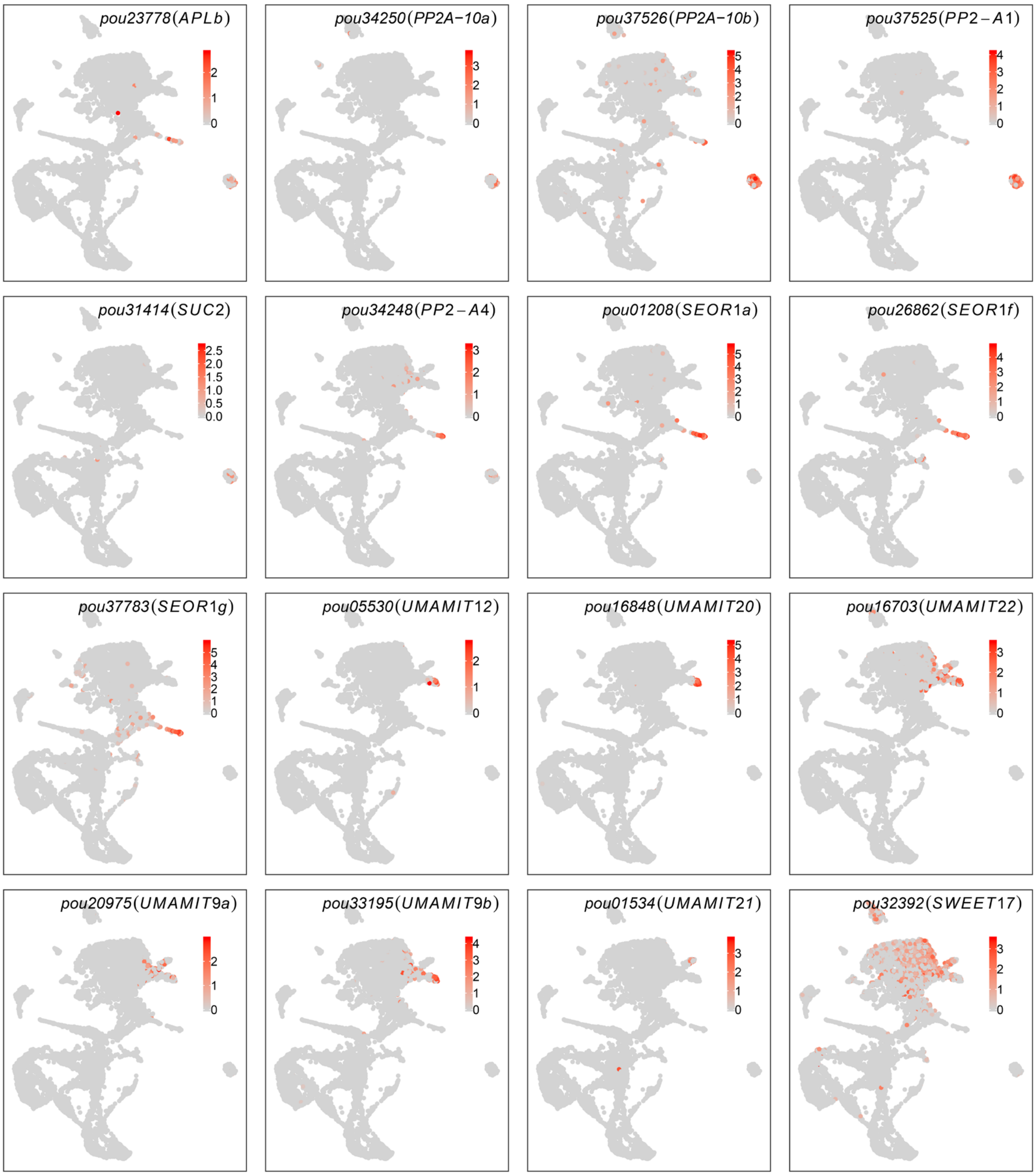

Fig. S9. Expression patterns of representative phloem-specific marker genes. The names in parentheses indicate the closest homologous genes in *Arabidopsis*, and lowercase letters indicate the numbers of multiple gene copies in *Populus alba* var. *pyramidalis*.

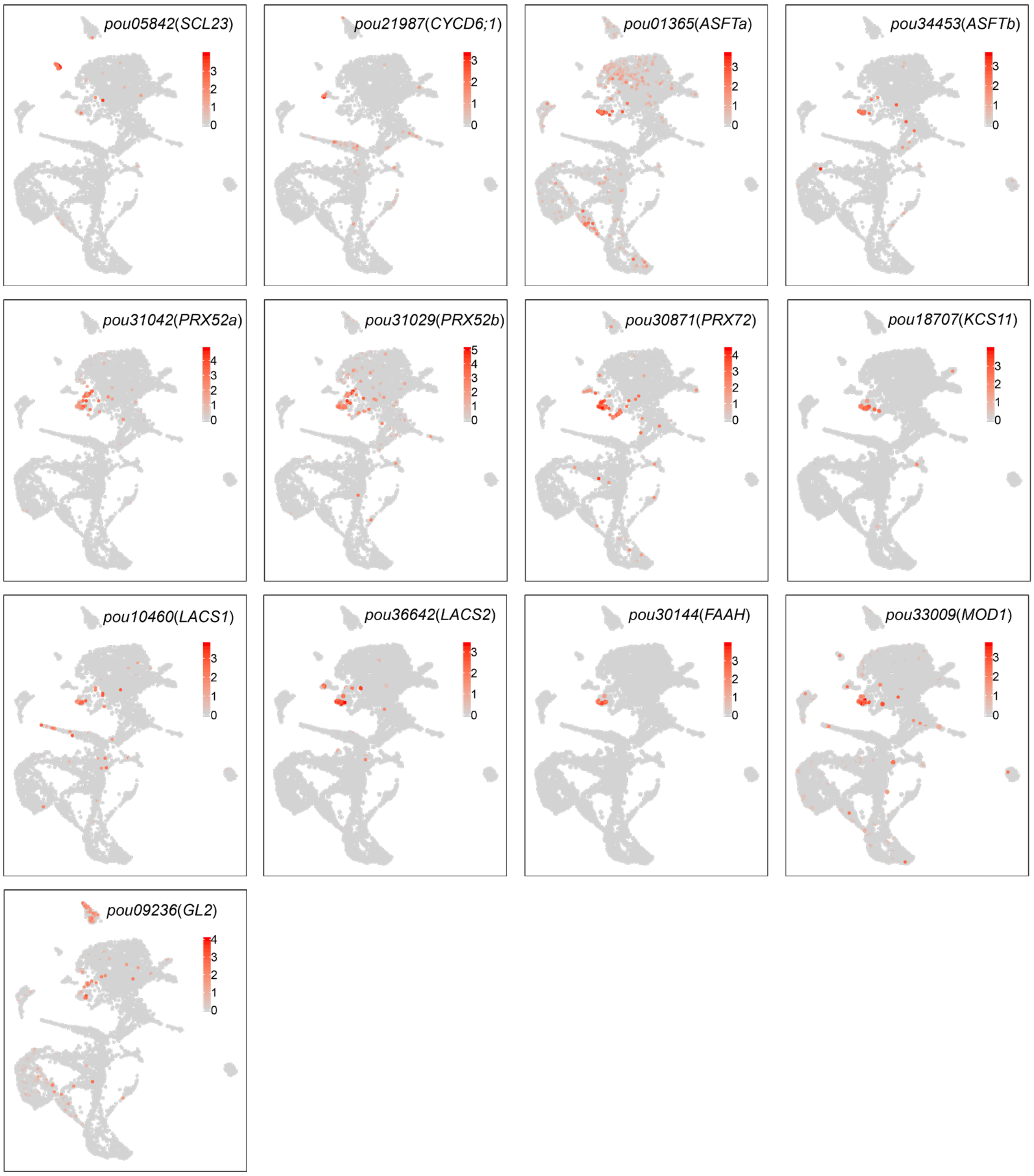

Fig. S10. Expression patterns of representative marker genes in cluster 7, 11, 12, 16 and 17. The names in parentheses indicate the closest homologous genes in *Arabidopsis*, and lowercase letters indicate the numbers of multiple gene copies in *Populus alba* var. *pyramidalis*.

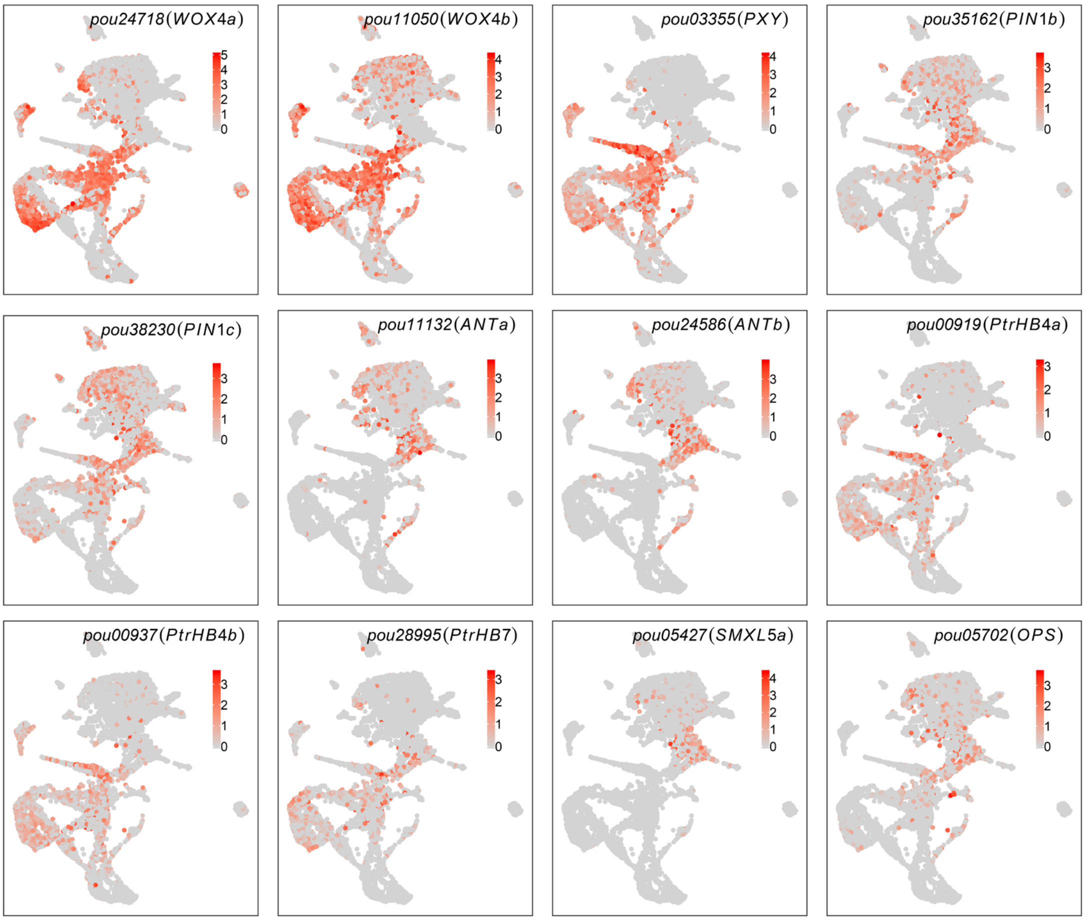

Fig. S11. Expression patterns of cambium marker genes. The names in parentheses indicate the closest homologous genes in *Arabidopsis*, and lowercase letters indicate the numbers of multiple gene copies in *Populus alba* var. *pyramidalis*.

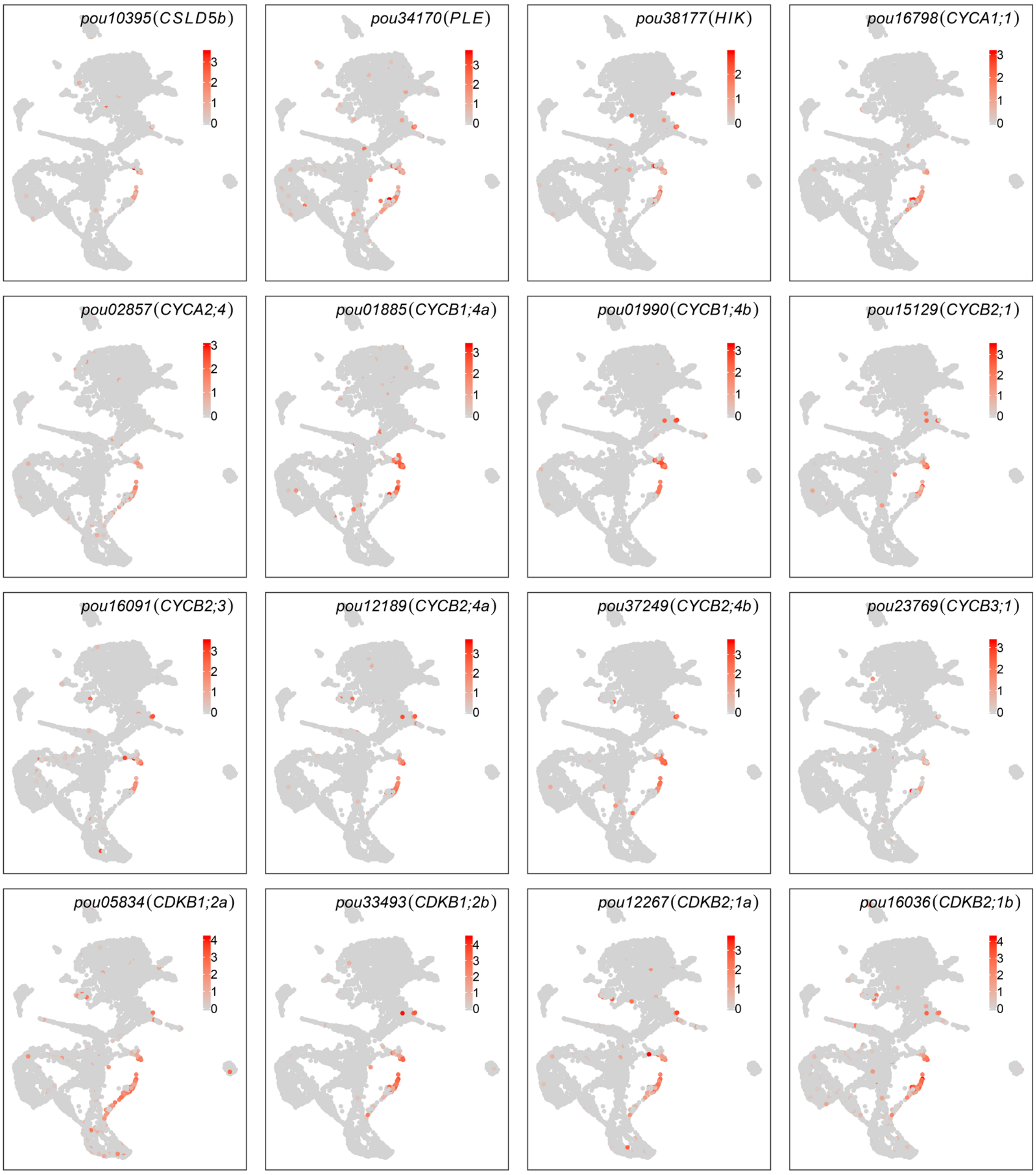

Fig. S12. Expression patterns of genes associated with mitosis and cell cycle. The names in parentheses indicate the closest homologous genes in *Arabidopsis*, and lowercase letters indicate the numbers of multiple gene copies in *Populus alba* var. *pyramidalis*.

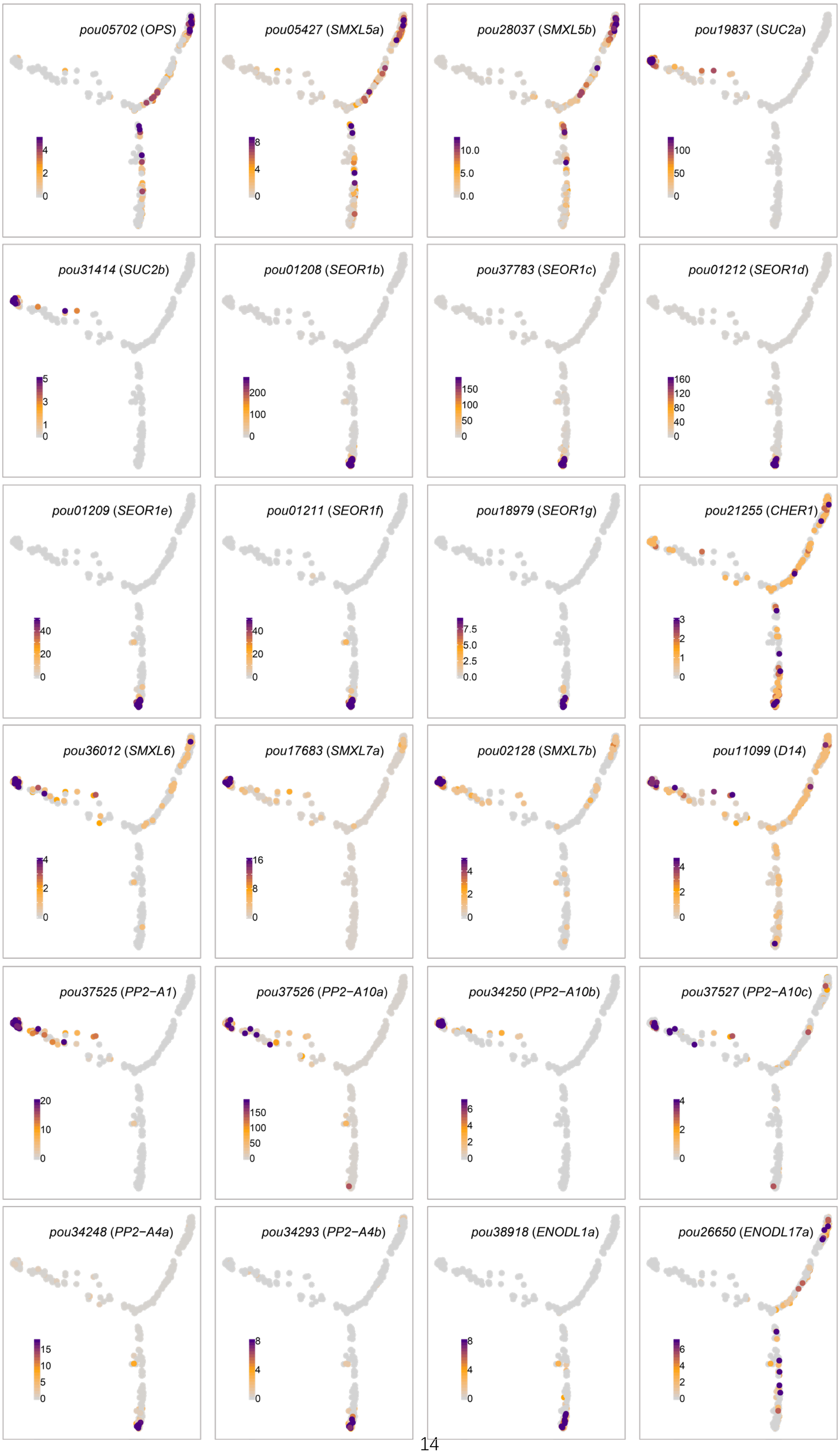
pou05702 (OPS)
pou05427 (SMXL5a)
pou28037 (SMXL5b)
pou19837 (SUC2a)
pou31414 (SUC2b)
pou01208 (SEOR1b)
pou37783 (SEOR1c)
pou01212 (SEOR1d)
pou01209 (SEOR1e)
pou01211 (SEOR1f)
pou18979 (SEOR1g)
pou21255 (CHER1)
pou36012 (SMXL6)
pou17683 (SMXL7a)
pou02128 (SMXL7b)
pou11099 (D14)
pou37525 (PP2−A1)
pou37526 (PP2−A10a)
pou34250 (PP2−A10b)
pou37527 (PP2−A10c)
pou34248 (PP2−A4a)
pou34293 (PP2−A4b)
pou38918 (ENODL1a)
pou26650 (ENODL17a)

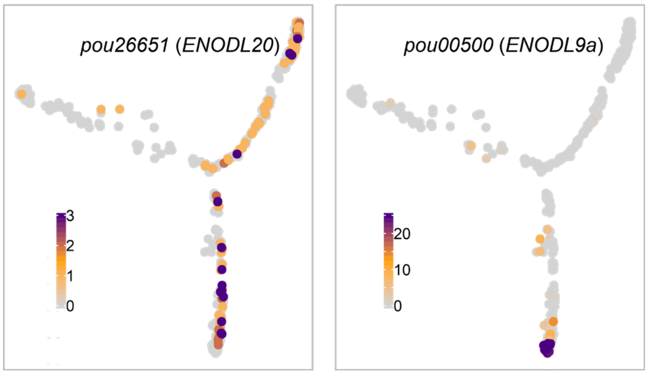

Fig. S13. Expression patterns of genes related to phloem development. The names in parentheses indicate the closest homologous genes in *Arabidopsis*, and lowercase letters indicate the numbers of multiple gene copies in *Populus alba* var. *pyramidalis*.

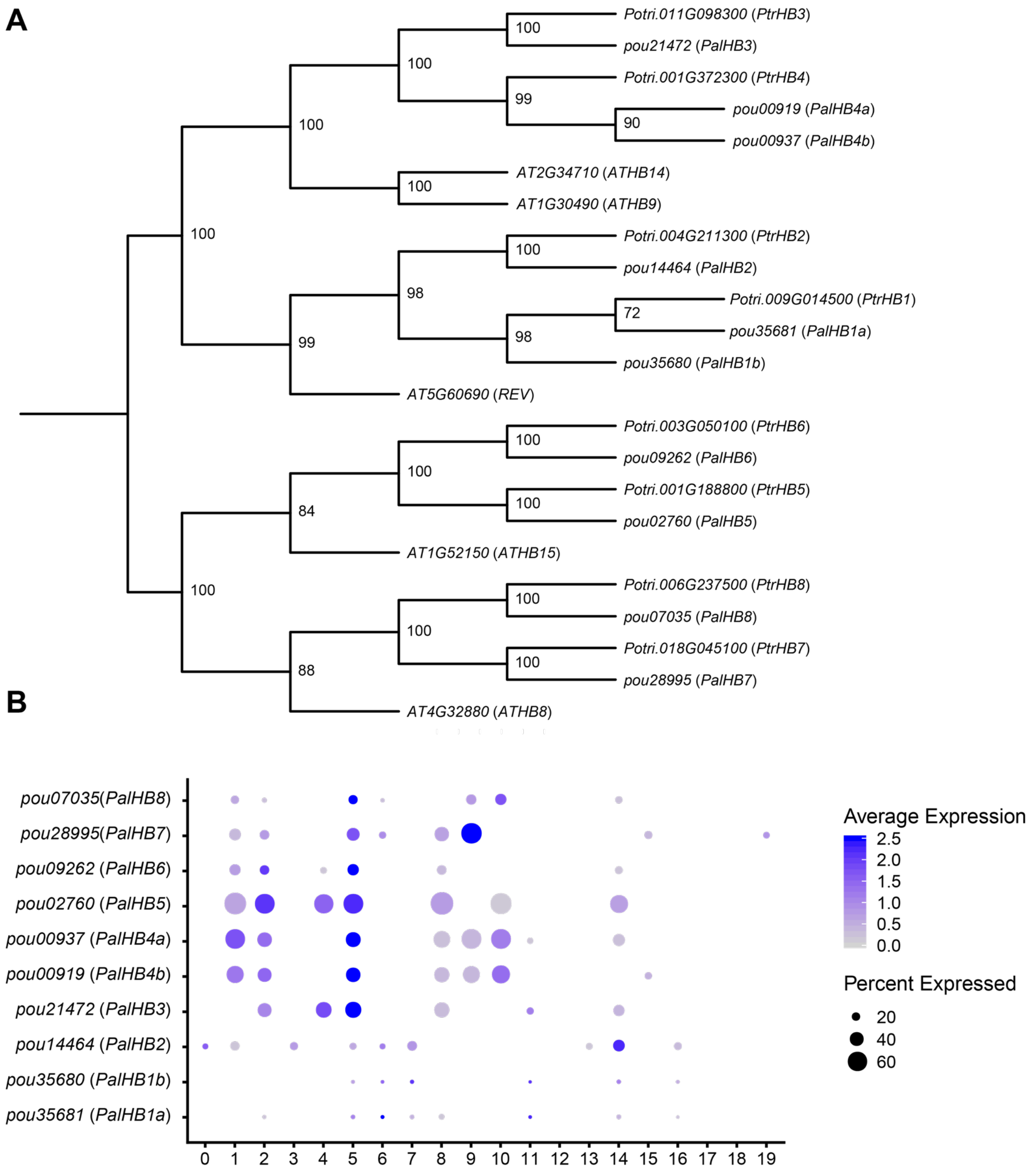

Fig. S14. Phylogenetic relationships and expression patterns of HD-ZIP III subfamily members. A. Phylogenetic relationship of HD-ZIP III subfamily of genes from *A. thaliana*, *P. trichocarpa* and *P. alba* var. *pyramidalis*. **B.** Expression patterns of *HD-ZIP III* genes in the 20 cell clusters identified in this study.

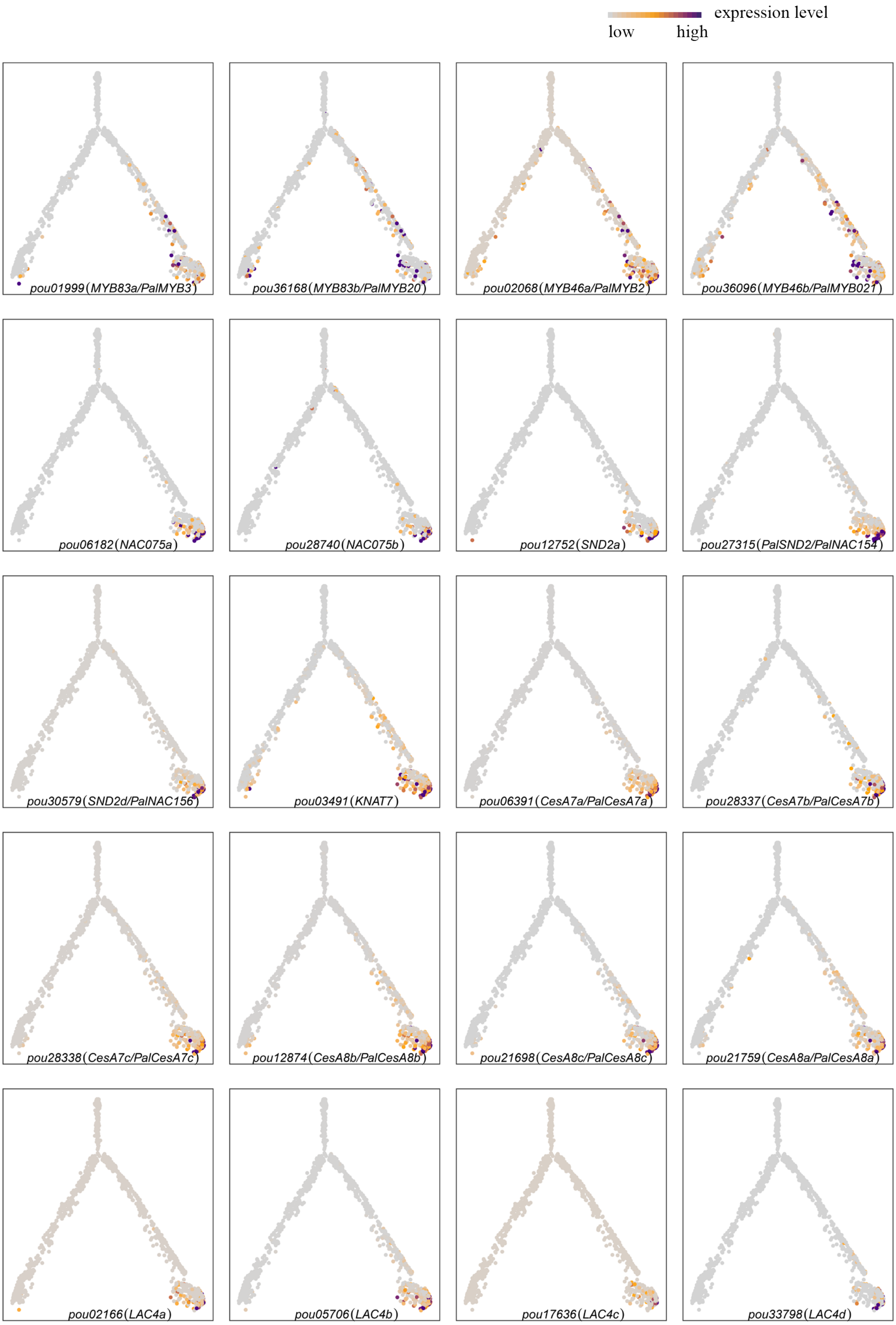
expression level
low
high
pou01999(MYB83a/PalMYB3)
pou36168(MYB83b/PalMYB20)
pou02068(MYB46a/PalMYB2)
pou36096(MYB46b/PalMYB021)
pou06182(NAC075a)
pou28740(NAC075b)
pou12752(SND2a)
pou27315(PalSND2/PalNAC154)
pou30579(SND2d/PalNAC156)
pou03491(KNAT7)
pou06391(CesA7a/PalCesA7a)
pou28337(CesA7b/PalCesA7b)
pou28338(CesA7c/PalCesA7c)
pou12874(CesA8b/PalCesA8b)
pou21698(CesA8c/PalCesA8c)
pou21759(CesA8a/PalCesA8a)
pou02166(LAC4a)
pou05706(LAC4b)
pou17636(LAC4c)
pou33798(LAC4d)

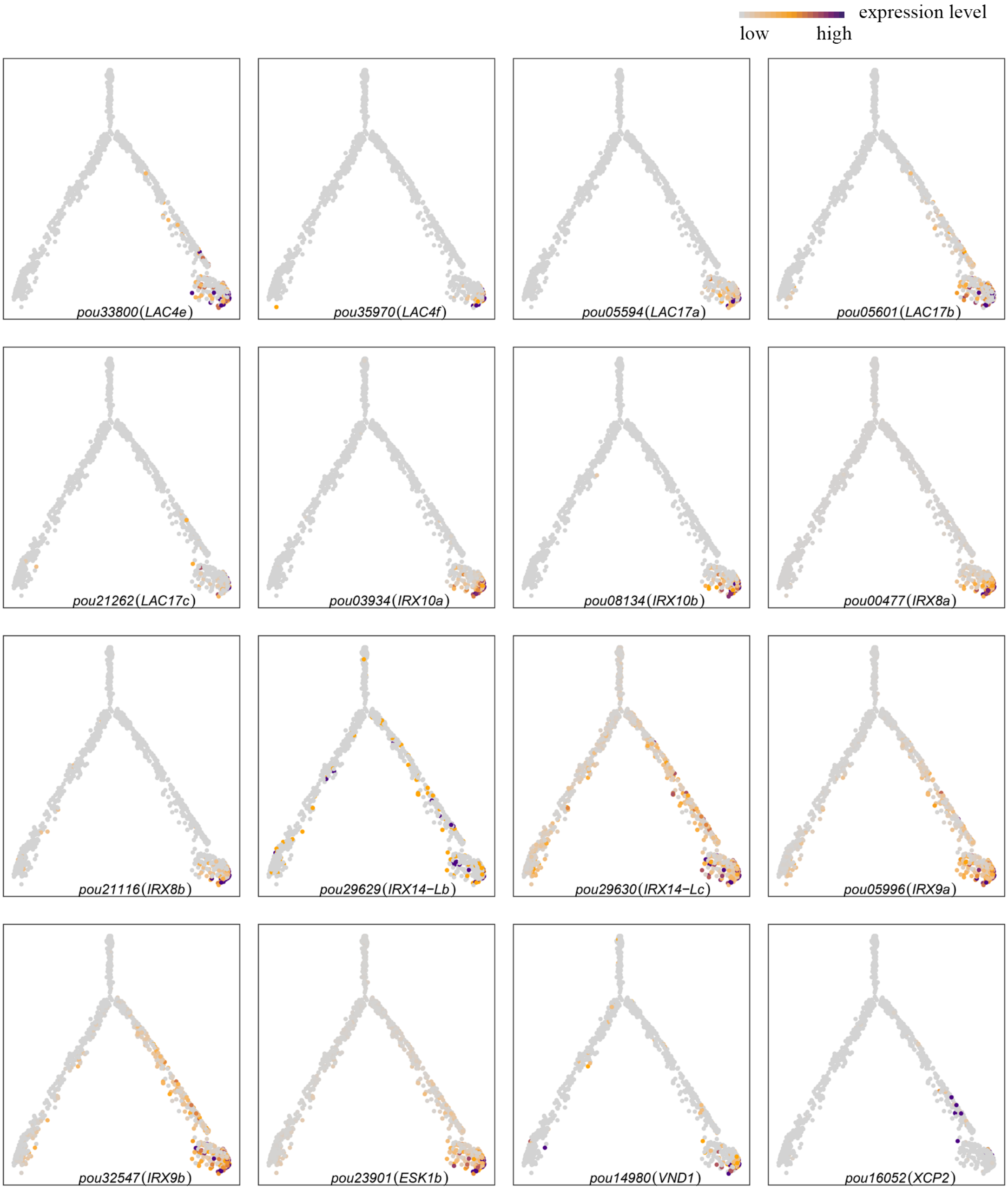

Fig. S15. Expression patterns of genes highly expressed in XSCW branch of the differentiation trajectory of xylem cells. The names in parentheses indicate the closest homologous genes in *Arabidopsis*, and lowercase letters indicate the numbers of multiple gene copies in *Populus alba* var. *pyramidalis*.

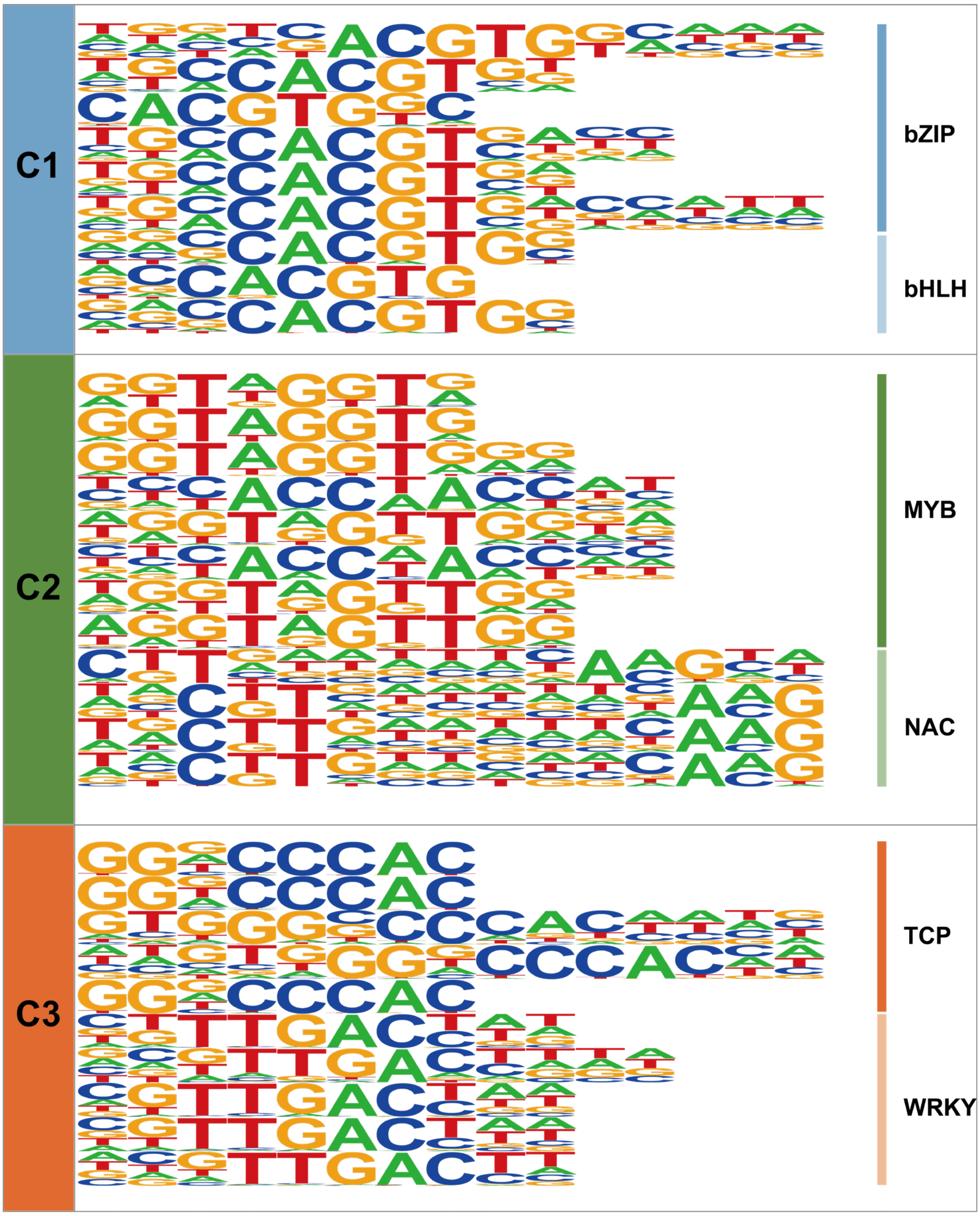

Fig. S16. Results of HOMER DNA-motif enrichment analyses using promoters (upstream 2000 bp) of branch-dependent genes identified in Figure 3C. Only binding motifs with q-values (adjusted by Benjamini-Hochberg) < 0.01 are shown.

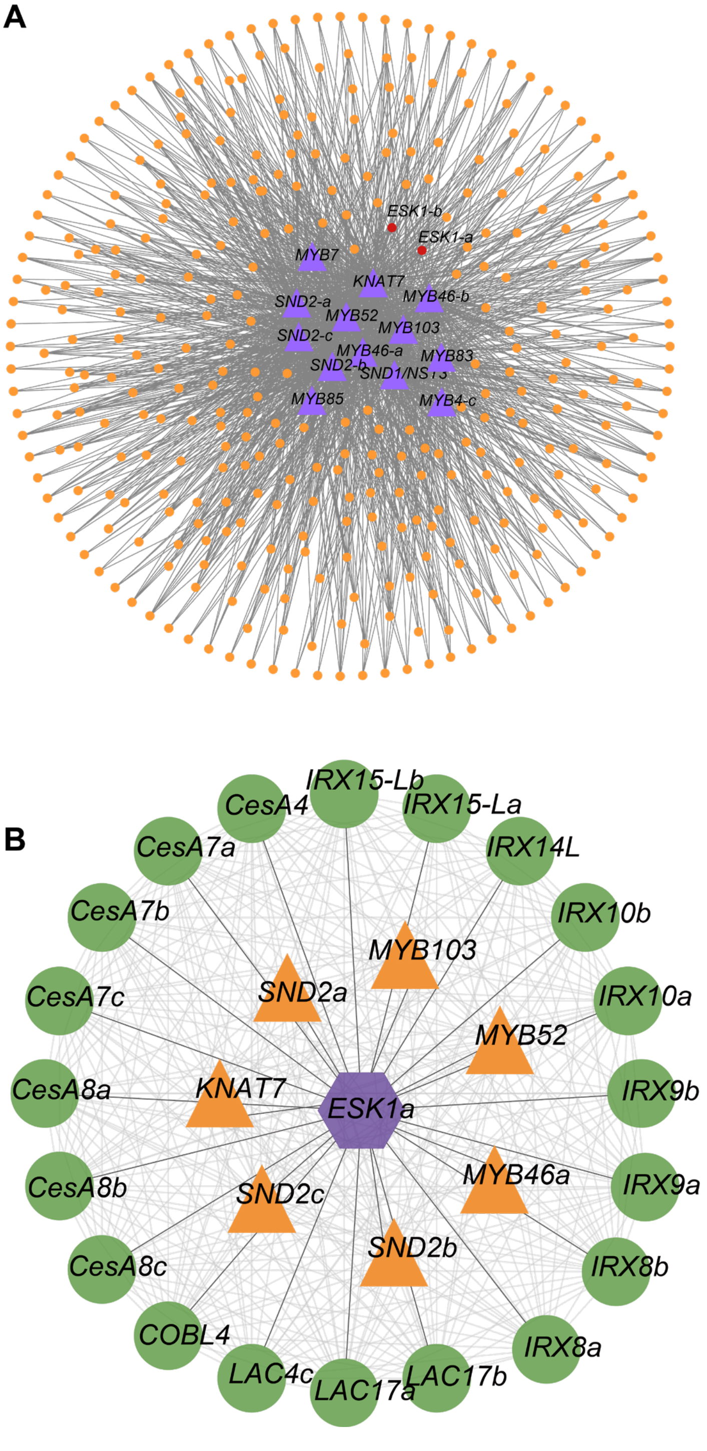

Fig. S17. Networks related to SCW formation. A. Co-expression network of key regulators during SCW formation and predicted candidate genes. Lines indicate Pearson correlation coefficients (> 0.5) for each pair of genes. **B.** Network constructed from Pearson correlations of SCW-associated protein-coding genes and transcription factor genes. Solid lines indicate correlations with $r > 0.5$. Dark solid lines indicate correlations with *ESK1a*.

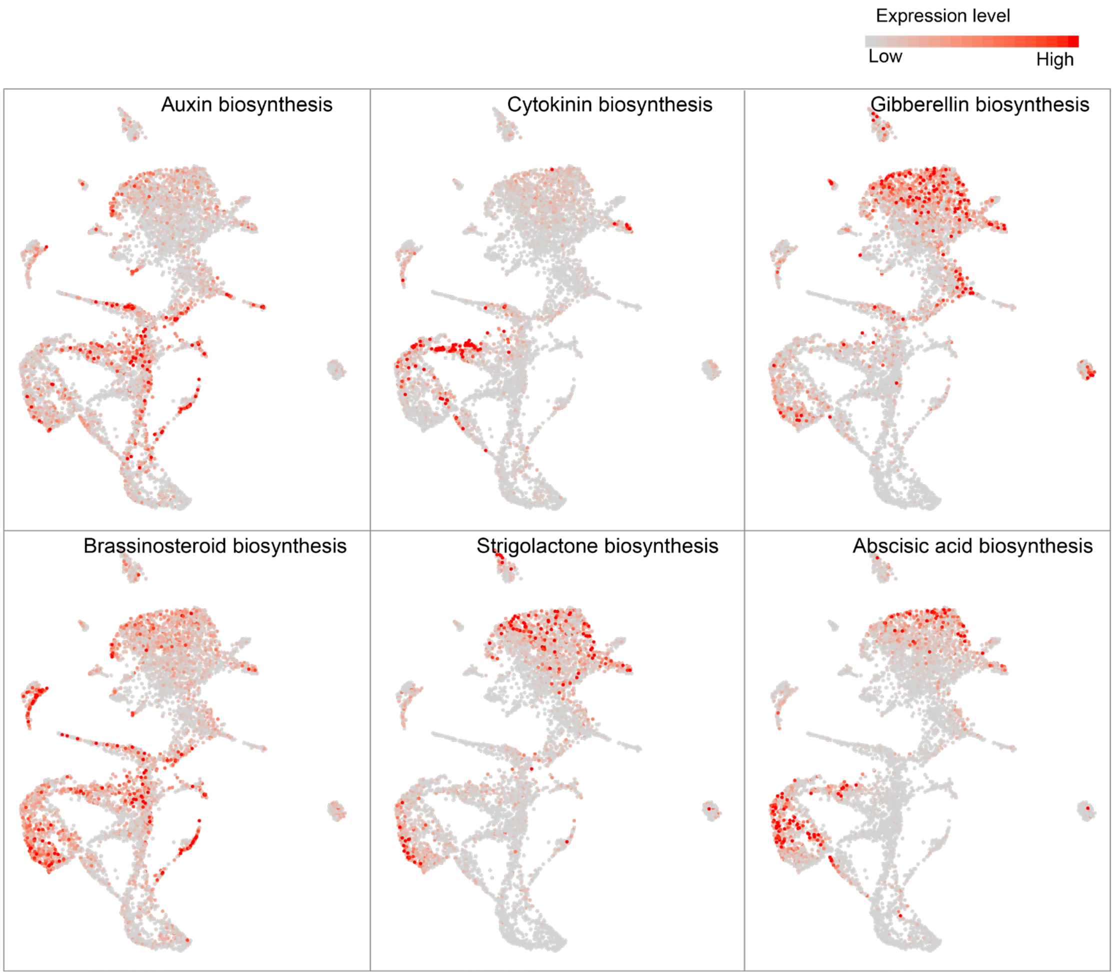

Fig. S18. UMAP visualization of expression patterns of genes responsive to auxin, cytokinin, gibberellin, brassinosteroid, strigolactone, and abscisic acid biosynthesis. The colors represent expression levels of these genes in individual cells.

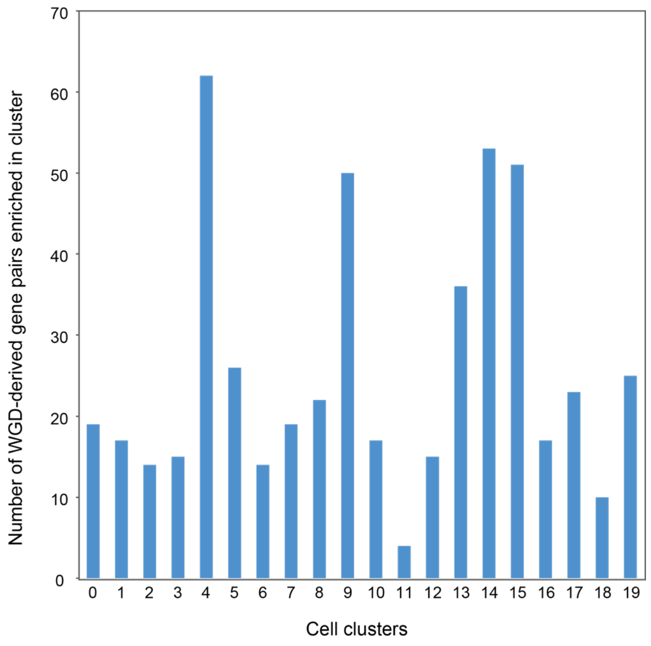

Fig. S19. Bar plot showing the number of overlaps between WGD-derived gene pairs and cell cluster-enriched genes.
