# Supplementary material for: Transcriptional landscape of highly lignified poplar stems at single-cell resolution
Source: Genome Biol. 2021 Nov 22;22:319. doi: 10.1186/s13059-021-02537-2 (PMC8607660; doi:10.1186/s13059-021-02537-2)
Supplement: Supplementary file 12 — Additional file 12. Review history. [file 13059_2021_2537_MOESM12_ESM.docx]

Review History

**First round of review**

**Reviewer 1**

**Are you able to assess all statistics in the manuscript, including the appropriateness of statistical tests used?**

Yes, and I have assessed the statistics in my report.

**Comments to author:**

Comments to the Author As far as I know, this is the first paper describing a cell atlas of poplar stems at the single-cell resolution. The application of scRNA-seq technology to study the differentiation trajectory of phloem and xylem in woody plants is in time and novel. In general, the bioinformatic analysis part including cell clustering, annotation and pseudotime inference meets the standard of the journal. However, these analyses does not yield novel and enough biological insights into phloem or xylem development. If authors can add more data related to this concern (for example, the last part of the manuscript confirms redundant roles of CesA7 and 8 genes, is it possible to get more examples based on their scRNA-seq dataset, and further prove it by the genome-editing tools?), I am happy to recommend it for publication. In addition, I have several points need to be clarified.

1. I am not sure whether all the harvested samples (bark and woody tissues) were efficiently protoplasted and captured. Could authors give some pictures to convince us? Is there any leftover (e.g. undigested tissues) after 2.5 hours of digestion?

2. Page 95. The rationale behind separating bark and woody tissues for scRNA-seq has been described. Any particular reason for this? Which cell clusters are overlapped between the bark and woody tissues (Fig. S3)? It is better to clarify which cell clusters belong to the bark and woody tissues respectively in Fig. 1B (please note that Fig S4 is not intuitive in nature).

3. I am not sure whether authors have mitigated the protoplasting and cell-cycle gene effects before cell clustering. If not, please refer to the method described in a recent paper from the Wang lab (Zhang et al., Dev Cell, 2021). I think this has become a standard for the scRNA-seq data analysis in plants.

4. The way by which the doublet is detected and removed is not described. Please provide more information on it. Particularly, we need to know the criteria which are used to define the parameters.

5. Introduction of the application of scRNA-seq in plants should be expanded. There are many research articles (e.g. Arabidopsis, Maize and rice) and reviews (e.g. one from the De Rybel lab on Annu Rev Plant Biol (2021); another from the Xu lab on Mol Plant (201)…) are available in PubMed.

6. The quality of the figures in the supplemental material is low (e.g. Fig. S2, low resolution and without annotations as in Fig. 1B). Wood: woody tissue.

**Reviewer 2**

**Are you able to assess all statistics in the manuscript, including the appropriateness of statistical tests used?**

There are no statistics in the manuscript.

**Comments to author:**

Review of the manuscript entitled "Transcriptional landscape of highly lignified poplar stems at single-cell resolution" by Chen et al., and submitted for publication to Genome Biology. Developing and applying an optimized methodology to isolate protoplasts from poplar stem, the authors conducted single-cell RNA-seq analyses to reveal the cell types composing the poplar stem and characterize the genes involved in cell wall biosynthesis. The authors identified 19 cell clusters and conducted a comprehensive annotation of the different cell clusters using known or orthologs to known marker genes. Besides, in situ hybridization confirmed the specificity of selected cell-type marker genes. Finally, transcriptomic trajectories were constructed to reveal the programs controlling the development of phloem and xylem cells. Overall, the manuscript is well written. Reference about each marker gene and their biological function is reported. This reviewer appreciated this information to give more biological information to the analyzed datasets. Below are several major and minor comments.

Major comments:

1. Previous work on Arabidopsis thaliana root demonstrated that cell wall components such as the presence of suberin can strongly affect the release of sub-population of protoplasts. Could the author discuss this observation in the context of the release of poplar bark and wood protoplasts? Specifically, how did the author estimate the release of protoplasts from all the cell types composing these tissues?

2. Line 102: Could the author clarify how many independent biological replicates were generated for bark and wood tissues? This reviewer expects that at least two independent replicates would be generated to ensure the reproducibility of the method, analysis, and results.

3. The last two chapters of the result section, especially "scRNA-seq predicts potential gene redundancy in poplar vascular development", are less informative. Regarding the last chapter, the analysis focuses on selected paralogs. A more comprehensive analysis is needed. Based on the datasets generated by the authors, this reviewer suggests the authors develop this more comprehensive analysis.

Minor comments:

1. Line 30: Could the authors mention that the cellular heterogeneity is based on their transcriptome?

2. Line 121: Could the authors provide fold-change and p-value information used to identify cluster-enriched genes?

3. Line 166: The authors mentioned that genes from the UMAMIT amino acid transporter family are preferentially expressed in clusters 17 and 18. This reviewer believes that these genes are mostly expressed in clusters 14 and 18.

4. Material and Method: The authors should justify the separation of both tissues. Using the same approach to isolate protoplast from both tissues. What was the advantage of separating the tissues before protoplast isolation?

**Reviewer 3**

**Are you able to assess all statistics in the manuscript, including the appropriateness of statistical tests used?**

Yes, and I have assessed the statistics in my report.

**Comments to author:**

This manuscript describes a single-cell RNA-seq dataset from poplar stems. The work provides new insight into the cell type diversity of wood, and identifies marker genes for phloem and xylem development. The study is timely and provide significant new insight into wood formation compared to previous bulk RNA-seq studies. Although another wood single-cell paper was published recently (4. August, https://onlinelibrary.wiley.com/doi/abs/10.1111/jipb.13159), I don't believe that this should compromise the novelty of the current paper, which was submitted before the other paper was published.

I have some suggestion:

1. I really think a table with the most prominent marker genes for each cell type would improve this paper and be an appreciated resource in the community. Now these markers are hidden in the text.

2. The authors should clarify if their materials were actually seedlings (meaning it comes from pollinated seed and would therefore be a mix of genotypes) or whether they mean clonal propagated cuttings from a single genotype.

3. The manuscript refers to three plants but it's unclear if there were three biological replicates of cells or if they combined material from those three and therefore actually have no biological replication at the sample level.

4. L. 105-106. 85% gene coverage of bulk RNA-seq. Is this based on number of genes alone or did you actually check that it is the same genes? At this stage in the manuscript it is unclear what data set was used for bulk RNA-seq. You should make it clear that this was also generated as part of this paper.

5. L. 106: "gene expression profiles" is confusing here. You correlate one expression value for each gene - across all genes. Use "gene expression values".

6. L. 236: How did you divide the cells into phloem and xylem cells? You later list the clusters you use for xylem but not for phloem (L. 292).

7. L. 561-565: In the interest of reproducibility, scripts producing results in this paper should be made available e.g. through GitLab.

8. L. 561: You should specify what background set of genes were used for enrichment tests. I would strongly advice using wood-expressed genes and not all genes in the genome. Also, what type of tests were used? What was the significance level? Did you correct for multiple testing?

9. L906 (Figure 1C): The violin plots need some more explanation.

10. L22: "on the activity of the vascular cambium"?

11. L25: Delete "involved"

12. L70: add "cell differentiation trajectories" (or something similar) to underline what is still missing? E.g. "However, the full diversity of cell types and cell differentiation trajectories in the vascular tissues of *woody plants have not yet been resolved."*

*13. L78: Change to "especially tissues of woody plants"?*

14. L128: Add "_and_ then…".

15. L439: delete "digital"

**Authors Response**

**Point-by-point responses to the reviewers’ comments:**

Reviewer #1:

As far as I know, this is the first paper describing a cell atlas of poplar stems at the single-cell resolution. The application of scRNA-seq technology to study the differentiation trajectory of phloem and xylem in woody plants is in time and novel. In general, the bioinformatic analysis part including cell clustering, annotation and pseudotime inference meets the standard of the journal. However, these analyses does not yield novel and enough biological insights into phloem or xylem development. If authors can add more data related to this concern (for example, the last part of the manuscript confirms redundant roles of CesA7 and 8 genes, is it possible to get more examples based on their scRNA-seq dataset, and further prove it by the genome-editing tools?), I am happy to recommend it for publication. In addition, I have several points need to be clarified.

*Reply: We appreciate the reviewer’s positive comments on our work. In the revised manuscript, we have responded to each of the comments point by point, and clearly pointed out the location of the revision. In addition, we have added a more comprehensive analysis to the functional redundancy section at the last part of the manuscript. We very much agree with the reviewer’s suggestion and hope that the genes we have detected can be further confirmed by genome-editing tools. However, it takes a long time to complete this experiment in poplar. Therefore, we have added more examples to the related figures and supplementary tables, hoping to be verified in future work. We also developed a web server (https://scu-populus.shinyapps.io/scRNAPal/) to facilitate the use of our scRNA-seq data.*

1. I am not sure whether all the harvested samples (bark and woody tissues) were efficiently protoplasted and captured. Could authors give some pictures to convince us? Is there any leftover (e.g. undigested tissues) after 2.5 hours of digestion?

*Reply: We have added a picture in Supplementary Figure 1 to show the tissues before and after digestion. We can find that our protocol captured most of the cell types, except for some cells with thickened secondary cell wall or cells with suberin, which were also pointed out in the discussion of the revised manuscript.*

2. Page 95. The rationale behind separating bark and woody tissues for scRNA-seq has been described. Any particular reason for this? Which cell clusters are overlapped between the bark and woody tissues (Fig. S3)? It is better to clarify which cell clusters belong to the bark and woody tissues respectively in Fig. 1B (please note that Fig S4 is not intuitive in nature).

*Reply: In woody plants, it is common to separate the bark and woody tissues for RNA sequencing, so that gene expression and cell types in different tissues (such as phloem and xylem) can be better studied, and the correlation analysis can be better performed with bulk RNA-seq data to ensure the reliability of our results. In addition, this strategy can also avoid the interference of cell debris or clumped cells digested by different tissues. In the revised manuscript, we have added information about the tissue sources of different cell clusters in Supplementary Figures 4 and 5.*

3. I am not sure whether authors have mitigated the protoplasting and cell-cycle gene effects before cell clustering. If not, please refer to the method described in a recent paper from the Wang lab (Zhang et al., Dev Cell, 2021). I think this has become a standard for the scRNA-seq data analysis in plants.

*Reply: In the revised manuscript, we reanalyzed our data using the method recommended by the reviewer to mitigate the effects of protoplasting and cell-cycle genes. We have added the description in the Methods section ‘Cell clustering and identification of cluster-enriched genes’ (Line 549-556). Thank you very much for your helpful suggestions to make our results more reliable.*

4. The way by which the doublet is detected and removed is not described. Please provide more information on it. Particularly, we need to know the criteria which are used to define the parameters.

*Reply: We have added the description of doublets detection and removal in the Methods section ‘scRNA-seq data pre-processing’ (Line 532-541).*

5. Introduction of the application of scRNA-seq in plants should be expanded. There are many research articles (e.g. Arabidopsis, Maize and rice) and reviews (e.g. one from the De Rybel lab on Annu Rev Plant Biol (2021); another from the Xu lab on Mol Plant (201)…) are available in PubMed.

*Reply: We have expanded the introduction of the application of scRNA-seq in plants and cited the articles mentioned by the reviewer in Line 79-85.*

6. The quality of the figures in the supplemental material is low (e.g. Fig. S2, low resolution and without annotations as in Fig. 1B). Wood: woody tissue.

*Reply: We have adjusted the resolution of all supplementary figures and added cell-type annotations in Supplementary Figure 3 of the revised manuscript.*

Reviewer #2:

Developing and applying an optimized methodology to isolate protoplasts from poplar stem, the authors conducted single-cell RNA-seq analyses to reveal the cell types composing the poplar stem and characterize the genes involved in cell wall biosynthesis. The authors identified 19 cell clusters and conducted a comprehensive annotation of the different cell clusters using known or orthologs to known marker genes. Besides, in situ hybridization confirmed the specificity of selected cell-type marker genes. Finally, transcriptomic trajectories were constructed to reveal the programs controlling the development of phloem and xylem cells.

Overall, the manuscript is well written. Reference about each marker gene and their biological function is reported. This reviewer appreciated this information to give more biological information to the analyzed datasets.

*Reply: We appreciate the reviewer’s positive comments on our work. In the revised manuscript, we have developed a web server (https://scu-populus.shinyapps.io/scRNAPal/) to further facilitate the use of our scRNA-seq data.*

Major comments:

1. Previous work on Arabidopsis thaliana root demonstrated that cell wall components such as the presence of suberin can strongly affect the release of sub-population of protoplasts. Could the author discuss this observation in the context of the release of poplar bark and wood protoplasts? Specifically, how did the author estimate the release of protoplasts from all the cell types composing these tissues?

*Reply: As recommended by the first reviewer, we have added a picture in Supplementary Figure 1 to show the tissues before and after digestion. It can be found that most of the cell types were captured by our protocol, except for some cells with thickened SCW or cells with suberin. In our scRNA-seq data, only a few cork cells (cluster 11) were successfully identified, which is consistent with the observations in Arabidopsis. We have pointed this out in the discussion of the revised manuscript (Line 458-464).*

2. Line 102: Could the author clarify how many independent biological replicates were generated for bark and wood tissues? This reviewer expects that at least two independent replicates would be generated to ensure the reproducibility of the method, analysis, and results.

*Reply: We very much agree with the reviewer’s suggestion that it is best to include independent replicates to ensure the reproducibility of our results. However, our data was generated early in the application of scRNA-seq technology in woody plants, the high price limits us to prepare another replicate. Therefore, we have done various analyses to determine the reliability of our data. The strong correlations between our scRNA-seq and bulk RNA-seq, the transcriptomes of cryosections of aspen stem, and in situ hybridization experiment of key marker genes all support the reliability of our results. In addition, some similar studies did not include independent replicates (such as Zhang et al. 2019, Molecular Plant 12:648-660; Liu et al. 2020, Molecular Plant 13:1178-1193; Liu et al. 2021, Plant Biotechnology Journal; Li et al. 2021, Journal of Integrative Plant Biology).*

3. The last two chapters of the result section, especially "scRNA-seq predicts potential gene redundancy in poplar vascular development", are less informative. Regarding the last chapter, the analysis focuses on selected paralogs. A more comprehensive analysis is needed. Based on the datasets generated by the authors, this reviewer suggests the authors develop this more comprehensive analysis.

*Reply: Thank you very much for your useful suggestions. We have added a more comprehensive analysis to the gene redundancy section according to the reviewer’s suggestion.*

Minor comments:

1. Line 30: Could the authors mention that the cellular heterogeneity is based on their transcriptome?

*Reply: We have clarified this description in the revised manuscript (Line 32-33).*

2. Line 121: Could the authors provide fold-change and p-value information used to identify cluster-enriched genes?

*Reply: The parameters used to identify cluster-enriched genes were provided in the Method section ‘Cell clustering and identification of cluster-enriched genes’ (Line 562-566). The fold-change and p-value information for each cluster-enriched gene were also provided in Supplementary Table 2.*

3. Line 166: The authors mentioned that genes from the UMAMIT amino acid transporter family are preferentially expressed in clusters 17 and 18. This reviewer believes that these genes are mostly expressed in clusters 14 and 18.

*Reply: We have updated the cell clustering information and expression of the UMAMIT amino acid transporter family can be observed in cluster 3 and 18 (Supplementary Figure 9).*

4. Material and Method: The authors should justify the separation of both tissues. Using the same approach to isolate protoplast from both tissues. What was the advantage of separating the tissues before protoplast isolation?

*Reply: As mentioned above, it is common to separate the bark and woody tissues for RNA sequencing in woody plants. The advantage of this strategy is that the gene expression and cell types in different tissues (such as phloem and xylem) can be better studied, and the correlation analysis can be better performed with bulk RNA-seq data to ensure the reliability of our results. In addition, this strategy can also avoid the interference of cell debris or clumped cells digested by different tissues. We have also added a picture in Supplementary Figure 1 to show the tissues before and after digestion, and the results showed that our strategy can capture most of the cell types.*

Reviewer #3:

This manuscript describes a single-cell RNA-seq dataset from poplar stems. The work provides new insight into the cell type diversity of wood, and identifies marker genes for phloem and xylem development. The study is timely and provide significant new insight into wood formation compared to previous bulk RNA-seq studies. Although another wood single-cell paper was published recently (4. August, https://onlinelibrary.wiley.com/doi/abs/10.1111/jipb.13159), I don't believe that this should compromise the novelty of the current paper, which was submitted before the other paper was published.

*Reply: We appreciate the reviewer’s positive comments on our work. In the revised manuscript, we have cited this article mentioned by the reviewer and developed a web server (https://scu-populus.shinyapps.io/scRNAPal/) to further facilitate the use of our data.*

I have some suggestion:

1. I really think a table with the most prominent marker genes for each cell type would improve this paper and be an appreciated resource in the community. Now these markers are hidden in the text.

*Reply: Thank you so much for your suggestion. We have added a table (Supplementary Table 5) to show the most prominent marker genes for each cell type identified in our study.*

2. The authors should clarify if their materials were actually seedlings (meaning it comes from pollinated seed and would therefore be a mix of genotypes) or whether they mean clonal propagated cuttings from a single genotype.

*Reply: The three poplars were clonal propagated cuttings from a single genotype and thus we have corrected the ‘seedlings’ to ‘plantlets’ in the revised manuscript (Line 104 and 492).*

3. The manuscript refers to three plants but it's unclear if there were three biological replicates of cells or if they combined material from those three and therefore actually have no biological replication at the sample level.

*Reply: We mixed material from three plantlets and then isolated protoplasts from them. They are not biological replication at the sample level. We have improved this unclear statement in the revised manuscript. As mentioned above, our data was generated early in the application of scRNA-seq technology in woody plants, the high price limits us to prepare another replicate. Therefore, we have done various analyses to determine the reliability of our data. The strong correlations between our scRNA-seq and bulk RNA-seq, the transcriptomes of cryosections of aspen stem, and in situ hybridization experiment of key marker genes all support the reliability of our results. In addition, some similar studies did not include independent replicates (such as Zhang et al. 2019, Molecular Plant 12:648-660; Liu et al. 2020, Molecular Plant 13:1178-1193; Liu et al. 2021, Plant Biotechnology Journal; Li et al. 2021, Journal of Integrative Plant Biology).*

4. L. 105-106. 85% gene coverage of bulk RNA-seq. Is this based on number of genes alone or did you actually check that it is the same genes? At this stage in the manuscript it is unclear what data set was used for bulk RNA-seq. You should make it clear that this was also generated as part of this paper.

*Reply: The bulk RNA-seq data were generated from undigested bark and woody tissues. The obtained clean reads were mapped to the same reference genome as scRNA-seq data (detailed in Line 568-580). Then we examined and compared the number of expressed genes in scRNA-seq and bulk RNA-seq (Transcripts Per Kilobase Million, TPM > 1).*

5. L. 106: "gene expression profiles" is confusing here. You correlate one expression value for each gene - across all genes. Use "gene expression values".

*Reply: Corrected.*

6. L. 236: How did you divide the cells into phloem and xylem cells? You later list the clusters you use for xylem but not for phloem (L. 292).

*Reply: The plant vascular tissue is mainly composed of xylem and phloem cells, which are produced by the inward and outward division of cambium, respectively. In this study, we first identified a variety of cell types using marker genes, such as sieve elements and companion cells from phloem (mainly from bark tissue), and xylem parenchyma cells, vessel and fiber cells from xylem (mainly from woody tissue). Therefore, we used these cell types to construct differentiation trajectory of the phloem and xylem respectively. The cell clusters used for phloem developmental trajectory were listed in the revised manuscript (Line 247-249).*

7. L. 561-565: In the interest of reproducibility, scripts producing results in this paper should be made available e.g. through GitLab.

*Reply: The link to the scripts we used in this study were provided in Line 613-614.*

8. L. 561: You should specify what background set of genes were used for enrichment tests. I would strongly advice using wood-expressed genes and not all genes in the genome. Also, what type of tests were used? What was the significance level? Did you correct for multiple testing?

*Reply: For GO enrichment, all genes, bark-expressed genes, and wood-expressed genes were selected as background sets in cluster-specific (Figure 1D), phloem-branch-dependent (Figure 2E) and xylem-branch-dependent (Figure 3D) analysis, respectively. P-value was obtained by Fisher’s exact test and adjusted by Benjamini-Hochberg false discovery rate. This information was provided in Line 614-618 of the revised manuscript.*

9. L906 (Figure 1C): The violin plots need some more explanation.

*Reply: Done.*

10. L22: "on the activity of the vascular cambium"?

*Reply: Corrected.*

11. L25: Delete "involved"

*Reply: Corrected.*

12. L70: add "cell differentiation trajectories" (or something similar) to underline what is still missing? E.g. "However, the full diversity of cell types and cell differentiation trajectories in the vascular tissues of *woody plants have not yet been resolved."*

*Reply: Added.*

*13. L78: Change to "especially tissues of woody plants"?*

*Reply: Corrected.*

14. L128: Add "_and_ then…".

*Reply: Corrected.*

15. L439: delete "digital"

*Reply: Corrected.*

**Second round of review**

**Reviewer 1**

Most of my concerns have been addressed. I am happy to recommend it for publication in Genome Biology.

**Reviewer 2**

The authors have addressed all my concerns.
